# Integrated Neurorehabilitation Including EEG-Neurofeedback for Unilateral Spatial Neglect After Right Thalamo-Mesencephalic Intracerebral Hemorrhage: A Case Report

**DOI:** 10.3390/neurolint18090167

**Published:** 2026-08-27

**Authors:** Anna Farella, Gianvito Lagravinese, Valerio Manippa, Paola Santacesaria, Rosanna Falcone, Maria Concetta Schiavariello, Pietro Fiore, Giorgia Francesca Scaramuzzi, Stefania De Trane, Paolo Taurisano

**Affiliations:** 1 Istituti Clinici Scientifici Maugeri IRCCS, Laboratory of Neuropsychology, Bari Institute, 70124 Bari, Italypaola.santacesaria@icsmaugeri.it (P.S.); 2 Department of Theoretical and Applied Sciences, eCampus University, 22060 Novedrate, Italy; valerio.manippa@uniecampus.it; 3 Istituti Clinici Scientifici Maugeri IRCCS, Neurorehabilitation Unit, Bari Institute, 70124 Bari, Italy; rosanna.falcone@icsmaugeri.it (R.F.); pietro.fiore@icsmaugeri.it (P.F.); stefania.detrane@icsmaugeri.it (S.D.T.); 4 Department of Physical and Rehabilitation Medicine, University of Foggia, 71122 Foggia, Italy; 5 Department of Translational Biomedicine and Neuroscience, University of Bari Aldo Moro, 70124 Bari, Italy; g.scaramuzzi6@phd.uniba.it (G.F.S.); paolo.taurisano@uniba.it (P.T.)

**Keywords:** visuospatial neglect, thalamic stroke, hemorrhagic stroke, EEG-based neurofeedback, qEEG, neurorehabilitation, spatial attention, single-case study

## Abstract

**Background/Objectives:** Unilateral Spatial Neglect (USN) is a disabling attentional syndrome occurring after cortical or subcortical stroke, including thalamic lesions. EEG-based neurofeedback (EEG-NF) may support cortical self-regulation, but evidence in spatial neglect remains limited. This case report describes the clinical, neuropsychological, functional, and electrophysiological (quantitative EEG, qEEG) evolution of a patient with USN after a right thalamic hemorrhagic stroke who underwent multidisciplinary rehabilitation including EEG-NF. **Methods:** A 70-year-old patient underwent a 3-week inpatient multidisciplinary rehabilitation program including physiotherapy, speech and swallowing therapy, conventional cognitive stimulation, and 15 adjunctive EEG-NF sessions. Baseline and post-treatment assessments included clinical, neuropsychological, neglect-specific, functional, affective, and resting-state qEEG measures. Individual change was examined using the Reliable Change Index when suitable psychometric parameters were available, together with published normative and clinical thresholds. **Results:** After rehabilitation, the Montreal Cognitive Assessment total raw score increased from 20 to 24, reaching the published minimal clinically important difference but not the minimal detectable change. The Frontal Assessment Battery increased from 8 to 12; after adjustment for age and education according to Italian normative data, both scores remained below the normative cut-off. Neglect-specific measures showed reduced spatial asymmetry, increased contralesional target detection, and a Catherine Bergego Scale reduction from 17 to 5, indicating a shift from moderate to mild ecological neglect. Functional independence improved, with reliable change in Functional Independence Measure (FIM) total and cognitive scores and Modified Barthel Index scores. Exploratory qEEG analyses showed condition-dependent spectral and connectivity changes, but findings were heterogeneous and hypothesis-generating. EEG-NF was completed without adverse events. **Conclusions:** The adjunctive EEG-NF protocol was completed as planned, with no adverse events. Clinical improvement cannot be attributed specifically to neurofeedback because all rehabilitation components were delivered concurrently. Controlled studies are needed to clarify its specific contribution and the durability of the observed improvements in post-stroke spatial neglect.

## 1. Introduction

Unilateral spatial neglect (USN) is a disabling neuropsychological syndrome commonly observed after stroke, characterized by a reduced ability to detect, orient toward, explore, or respond to stimuli located in the space contralateral to the brain lesion. This condition cannot be explained solely by primary sensory or motor deficits and may involve perceptual, attentional, motor-intentional, representational, and behavioral components. From a clinical perspective, USN is particularly relevant because it negatively affects functional recovery, autonomy in activities of daily living, and rehabilitation outcomes after stroke [1]. With a prevalence reaching up to 50% after a right-hemisphere stroke, USN remains a primary driver of long-term disability and poor rehabilitation prognosis [2].

USN is most frequently associated with right-hemisphere lesions and reflects disruptions of cortical fronto-parietal and temporo-parietal networks involved in spatial orienting and attentional control [2]. However, neglect should not be considered a purely cortical syndrome. Increasing evidence supports a network-based model of spatial attention. In this model, cortical and subcortical structures jointly contribute to attention, arousal, behavioral regulation, and interhemispheric balance. Within this model, the USN clinical phenotype is thought to arise from disrupted connectivity between fronto-parietal attentional systems and vigilance networks [2], and its clinical recovery relies heavily on restoring balanced interhemispheric dynamics [3].

In this framework, the thalamus plays a role not only as a relay station but also as an active node within cortico-subcortical circuits supporting attention and spatial exploration. Recent evidence suggests that USN can emerge following isolated right thalamic lesions, likely due to the aforementioned functional disruption of broader network nodes [4]. Within these subcortical circuits, lesions involving hubs such as the mediodorsal nucleus can impair goal-directed attention and cognitive flexibility. This suggests that disruption of thalamo-cortical loops compromises executive control [5].

Rehabilitation of USN remains challenging. Several approaches have been proposed, including visual scanning training, prism adaptation, limb activation, optokinetic stimulation, non-invasive brain stimulation, virtual reality-based interventions, and compensatory strategies [1]. Nevertheless, treatment response is variable. Patients with complex post-stroke presentations often require multidisciplinary programs that integrate motor, cognitive, language, and functional interventions. This is particularly relevant in real-world neurorehabilitation settings, where clinical improvement usually reflects the interaction of multiple therapeutic components rather than the isolated effect of a single technique. Current strategies often combine top-down and bottom-up approaches, such as visual scanning, compensatory strategy training, and technology-assisted interventions, to promote environmental exploration and functional adaptation [2].

In recent years, EEG-based neurofeedback has been proposed as a potential adjunctive approach for post-stroke neurorehabilitation. Neurofeedback provides patients with real-time information about their own brain activity, allowing them to progressively learn to modulate specific neurophysiological patterns through implicit or explicit self-regulation strategies. Concerning spatial neglect, the available EEG-NF evidence remains limited and has mainly targeted posterior alpha activity. Ros et al. [6] trained five patients with left visuospatial neglect after right-hemisphere stroke across six daily sessions of P4 alpha downregulation. Neurofeedback control improved progressively, and restoration of the resting alpha-rhythm dynamic range was associated with better visuospatial search performance [6]. Saj et al. [7] subsequently applied a single 30 min P4 alpha downregulation session to four patients in the acute phase after a right-hemisphere stroke. All patients reduced posterior alpha power during training and showed immediate improvement in neglect measures. However, the difference between pre-treatment and 1-week follow-up was not significant [7]. A subsequent perspective integrating EEG and rt-fMRI neurofeedback findings emphasized that larger controlled studies with longer follow-up are still required before the effectiveness of these approaches can be established [8]. 

Preliminary studies have primarily targeted posterior parietal networks, either to modulate alpha rhythms [6] or to reduce interhemispheric imbalance through brain–computer interface (BCI) approaches, which translate brain activity patterns into control signals or feedback to support rehabilitation [3,9]. By contrast, central electrode sites (e.g., Cz) may represent an alternative target for supporting broader attentional stability and sensorimotor regulation. In a multimodal rehabilitation context, a central electrode placement may help optimize cortical vigilance and sensorimotor regulation rather than aiming to directly influence deep subcortical structures.

This case report describes the clinical, neuropsychological, and functional evolution of a patient with USN following a right thalamo-mesencephalic hemorrhagic stroke who underwent an intensive multidisciplinary rehabilitation program including physiotherapy, speech and language therapy, conventional cognitive stimulation, and adjunctive EEG-NF. The primary objective was to document the feasibility, tolerability, and clinical evolution of the patient throughout treatment, without drawing causal conclusions regarding the isolated therapeutic impact of the neurofeedback protocol. A secondary exploratory objective was to characterize pre–post changes in resting-state spectral power and sensor-level functional connectivity, the latter assessed using the weighted Phase Lag Index. These measures were treated as potential neurophysiological correlates of the patient’s clinical evolution during the integrated rehabilitation program. These electrophysiological analyses were descriptive and were not intended to test a treatment-specific neurophysiological mechanism.

## 2. Materials and Methods

### 2.1. Case Presentation

On 18 March 2026, a 70-year-old man was admitted to the emergency department following the acute onset of neurological symptoms consistent with a cerebrovascular event.

Symptoms developed abruptly during lunch with his family: the patient developed sudden weakness in his left hand, rapidly followed by loss of trunk control with left-sided deviation and drooping of the corner of the mouth. The regional emergency system was activated, the stroke pathway was triggered, and the patient was transferred to the Stroke Unit.

He had completed 11 years of education and was retired. His vascular risk profile was notable: untreated hypertension, hypercholesterolemia, hyperhomocysteinemia due to folate deficiency, and hyperuricemia. He was a current smoker (approximately 10 cigarettes per day) and reported moderate alcohol consumption (one glass of wine with meals). A family history of stroke was present (father deceased from stroke). He reported no allergies and was not taking any medication.

On admission, the patient was alert, cooperative, and able to answer questions. Neurological examination revealed left homonymous hemianopia, moderate dysarthria, left facio-brachio-crural hemiplegia, and left hemianesthesia.

An emergency non-contrast cranial computed tomography scan (CT) showed an intraparenchymal hematoma in the right thalamus with compression of the third ventricle. Serial imaging confirmed hemorrhagic stability, while contrast-enhanced CT on 19 March better delineated perifocal edema around the right thalamo-mesencephalic hemorrhagic focus, without significant interval change.

**Figure 1:**
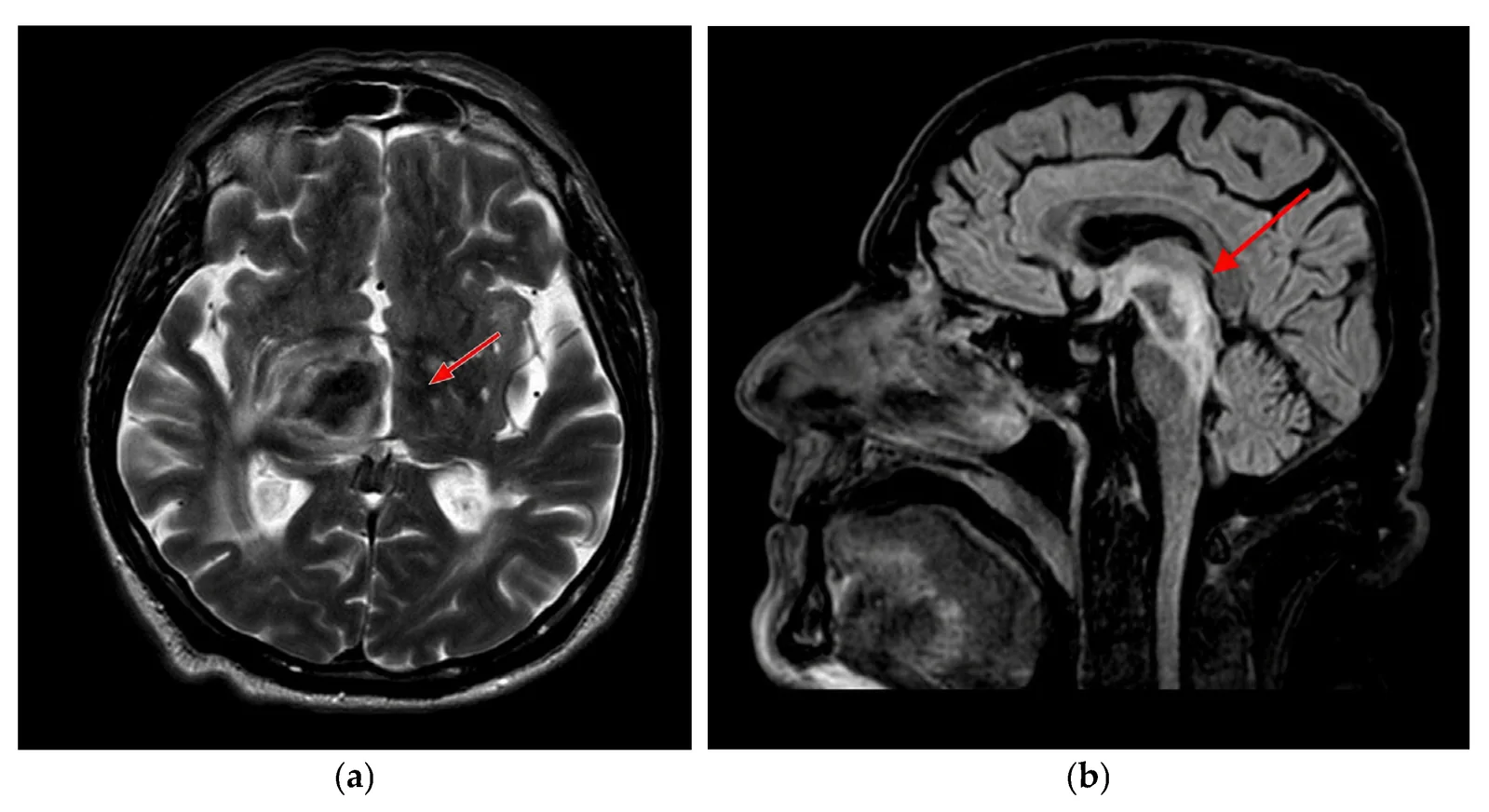
Representative brain MRI images: (**a**) Axial T2-weighted image showing a right thalamo-mesencephalic lesion with heterogeneous signal intensity and surrounding edema. (**b**) Sagittal FLAIR image showing the cranio-caudal extension of the lesion and its involvement of the diencephalic–mesencephalic region. Red arrows indicate the right thalamo-mesencephalic hemorrhagic lesion.

The patient was closely monitored throughout his stay in the Stroke Unit. Brain Magnetic Resonance Imaging (MRI) on 23 March (Figure 1) confirmed a right thalamo-mesencephalic hemorrhagic focus with perifocal edema extending to the superior cerebellar peduncle, pons, and right optic tract. The hematoma had opened into the ventricular system, with hemorrhagic traces visible particularly in the left occipital horn. Furthermore, a less recent hemorrhagic focus was identified in the right temporo-basal region adjacent to the lateral ventricle, with a hemosiderin rim and minimal peripheral enhancement. Leukoaraiosis of the periventricular white matter was noted, and Susceptibility Weighted Imaging (SWI) sequences revealed punctate hemosiderin foci in the bilateral parietal subcortical regions and median pons, also raising the possibility of capillary telangiectasia. Mild cortical atrophy was reflected in the widening of cortical sulci and basal cisterns.

By the time of discharge on 26 March, the patient had clinically stabilized, though meaningful deficits persisted: moderate left hemiparesis, left hemihypoesthesia, and gaze deviation consistent with a right third cranial nerve deficit. Intensive post-acute inpatient rehabilitation was therefore deemed necessary. The patient was transferred to the Neuromotor Rehabilitation Unit of the IRCCS Maugeri Institute in Bari to begin a structured program aimed at motor recovery. At admission to inpatient neurorehabilitation, the patient was alert but drowsy, cooperative, and partially oriented. Reduced awareness of his motor and functional deficits, increased distractibility, and limited spontaneous engagement were observed. Neuromotor examination showed left facio-brachio-crural hemiparesis, with minimal voluntary activation of the left upper limb and more preserved recruitment in the left lower limb. Muscle strength ranged from 0 to 2 on the left upper limb and from 2 to 3 on the left lower limb, whereas right-sided strength was relatively preserved. Left-sided tactile, nociceptive, and proprioceptive hypoesthesia was present. Additional findings included a left central facial deficit, right eyelid ptosis, limitation of upward gaze, abnormalities of saccadic movements and convergence, left homonymous hemianopia, and mild dysarthria.

Head and trunk control and sitting balance were severely impaired. Standing and gait were not initially achievable, and the Timed Up and Go test was not executable. The patient required complete assistance with transfers, personal hygiene, postural transitions, and basic activities of daily living. Baseline functional scores were 17/91 on the FIM motor subscale, 19/35 on the FIM cognitive subscale, 36/126 on the FIM total score, and 5/100 on the Modified Barthel Index. Dysphagia predominantly affecting the oral phase was also present.

**Figure 2:**
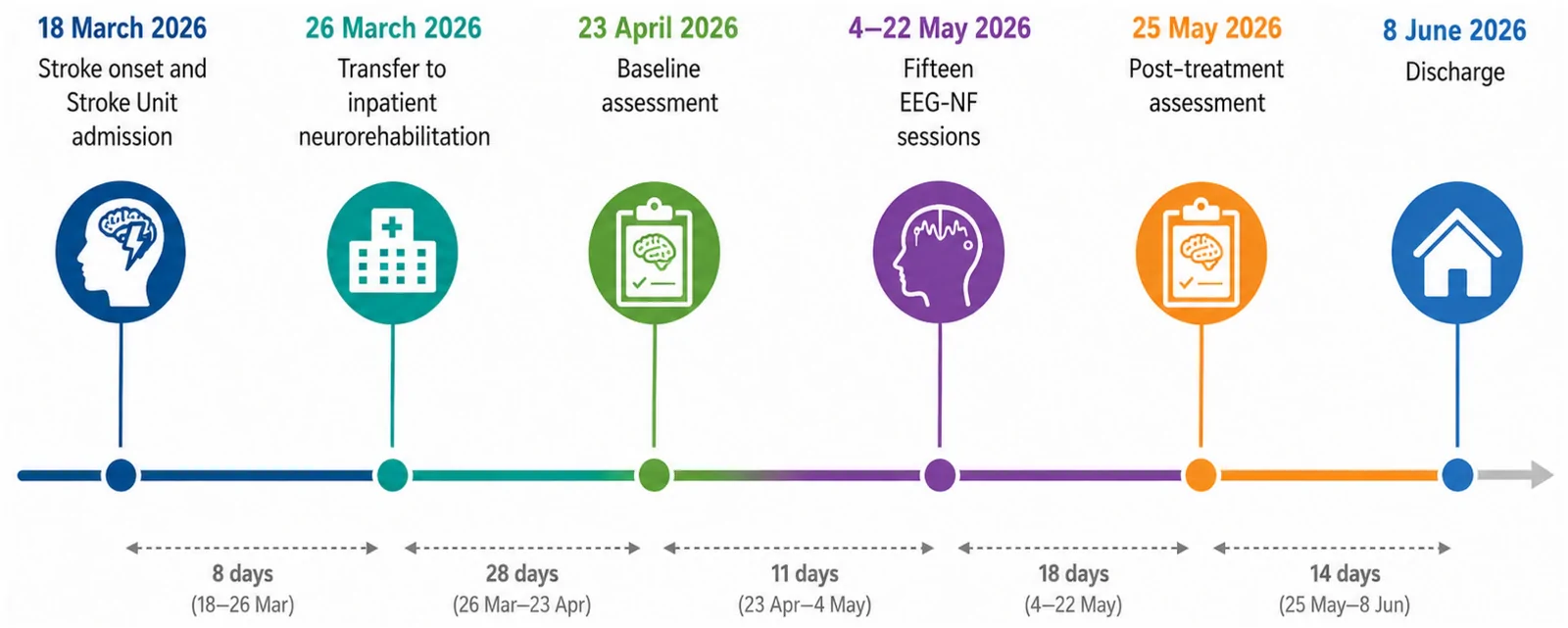
Clinical and rehabilitation timeline. The timeline summarizes stroke onset and Stroke Unit admission on 18 March 2026; transfer to inpatient neurorehabilitation on 26 March 2026; baseline multidimensional assessment on 23–24 April 2026, including clinical and neuropsychological assessment on 23 April and resting-state EEG acquisition on 24 April; completion of 15 EEG-NF sessions between 4 and 22 May 2026; post-treatment multidimensional assessment on 25 May 2026; and discharge on 8 June 2026.

At the baseline neuropsychological assessment on 23 April 2026, the patient appeared drowsy but arousable, cooperative, and partially oriented in space and time. Psychomotor slowing, reduced sustained attention, and limited awareness of motor and functional deficits were evident. Speech was mildly dysarthric but intelligible, and verbal comprehension was sufficiently preserved for assessment. Severe left USN involved personal, peripersonal, and extrapersonal space and was characterized by markedly asymmetric exploration, systematic omission of left-sided stimuli, impaired reading and visuoconstructive performance, and the need for continuous external facilitation during testing and functional activities. The patient remained hospitalized until discharge on 8 June 2026. The main clinical and rehabilitation time points are summarized in Figure 2.

### 2.2. Multidisciplinary Rehabilitation Program 

The study was conducted at the Neurorehabilitation Unit of ICS Maugeri IRCCS, Bari, Italy. The patient was admitted for intensive post-acute neurorehabilitation following a right thalamic hemorrhagic stroke and presented with clinically significant USN, together with associated motor, functional, and cognitive difficulties.

Before treatment initiation, the patient underwent a multidimensional baseline evaluation, including neuromotor and functional assessment, neuropsychological testing with neglect-specific measures, and resting-state quantitative EEG (qEEG) recording. These assessments were used to characterize the patient’s initial clinical profile and to establish a pre-intervention baseline across behavioral, cognitive, electrophysiological, and functional domains. 

The patient received an integrated multidisciplinary rehabilitation program including physiotherapy, speech and swallowing therapy, conventional cognitive stimulation, and adjunctive EEG-based neurofeedback (EEG-NF). Physiotherapy targeted postural control, balance, progressive mobilization, and gait recovery; speech and swallowing therapy addressed dysphagia, autonomous feeding, and communication; and cognitive rehabilitation focused on visual exploration, sustained attention, contralesional awareness, and compensatory strategies for USN. EEG-NF was incorporated as an adjunctive intervention aimed at supporting cortical self-regulation and attentional engagement. The frequency, duration, and principal objectives of each rehabilitation component are summarized in Table 1, while the EEG-NF protocol is described in the EEG-Neurofeedback Protocol subsection and Table 2.

#### EEG-Neurofeedback Protocol

EEG-NF was administered using the ProComp5 system with BioGraph Infiniti software, version 6.9.0.0 (Thought Technology, Montreal, QC, Canada). Electrode placement followed the international 10–20 system, with the active electrode at Cz and reference and ground electrodes placed at the left and right ears, respectively. Electrode impedance was maintained below 5 kΩ.

The individualized protocol reinforced activity in the 12–15 Hz sensorimotor rhythm (SMR) band while inhibiting theta (4–7.5 Hz) and high-beta (22–26 Hz) activity. The patient completed 15 sessions over 3 weeks (5 sessions/week), each lasting approximately 35 min. Real-time visual and auditory feedback was delivered through a gamified interface, and reinforcement thresholds were adaptively adjusted by the clinician to maintain engagement and an appropriate level of task difficulty.

All sessions were supervised, with monitoring of signal quality, artifacts, fatigue, discomfort, reduced engagement, and adverse events. EEG-NF was administered as an adjunctive component of the multidisciplinary rehabilitation program rather than as a stand-alone treatment. Complete protocol parameters are summarized in Table 2.

### 2.3. Outcome Measures

Baseline clinical, neuropsychological, and functional assessments were conducted on 23 April 2026, and the baseline resting-state EEG was acquired on 24 April 2026. Post-treatment clinical, neuropsychological, functional, and resting-state EEG assessments were conducted on 25 May 2026. Outcomes were organized into five domains: (i) treatment feasibility and tolerability; (ii) cognitive and affective functioning; (iii) neglect-specific and ecological functioning; (iv) neuromotor and functional independence; and (v) exploratory electrophysiological outcomes.

The primary clinical outcomes were changes in neglect-specific performance, ecological neglect-related disability, functional independence, and EEG-NF feasibility and tolerability. Secondary outcomes included global cognitive functioning, executive-attentional performance, affective symptoms, gait feasibility, and exploratory resting-state qEEG measures.

#### 2.3.1. Clinical, Neuropsychological, Neglect-Specific, and Functional Assessment

Global cognitive functioning was assessed using the Montreal Cognitive Assessment (MoCA). Executive-attentional functioning was examined using the Frontal Assessment Battery (FAB) [10], Clock Drawing Test [11], Digit Span Forward and Backward, phonemic and semantic verbal fluency, the Italian short Stroop Test [12], and the Arrow Stroop Test. Affective symptoms were assessed using the anxiety and depression subscales of the Hospital Anxiety and Depression Scale (HADS) [13,14].

Neglect-specific assessment included the spatial asymmetry measure of the Oxford Cognitive Screen [15,16]; the Bells Cancellation Test and Letter Cancellation Test, including detected targets and Center of Cancellation; the Timed Neglect Test; the Baking Tray Task; star cancellation; Diller letter cancellation; article reading; and object search in extrapersonal space. Ecological manifestations of neglect during activities of daily living were assessed using the Catherine Bergego Scale [17].

Neuromotor status was characterized through clinical examination of muscle strength, voluntary motor control, somatosensory functioning, cranial nerves, postural control, balance, and gait. Functional independence was assessed using the Functional Independence Measure (FIM), including total, motor, and cognitive scores, and the Modified Barthel Index. Mobility and gait feasibility were assessed using the Timed Up and Go test whenever executable.

EEG-NF feasibility was evaluated according to the number of completed sessions relative to the 15 sessions planned, treatment adherence, and the occurrence of treatment-related interruptions. Tolerability was assessed through clinical monitoring during and immediately after each session for fatigue, discomfort, headache, dizziness, nausea, agitation, reduced engagement, or sustained worsening of neurological or cognitive symptoms. Any adverse event and its possible relationship with treatment were recorded.

#### 2.3.2. EEG Acquisition and Preprocessing

Resting-state EEG was recorded at baseline and post-treatment using a Mitsar-201 system (Mitsar Co. Ltd., St. Petersburg, Russia) with 19 Ag/AgCl electrodes positioned according to the international 10–20 system. Approximately 6.3 min of EEG were acquired during both eyes-open (EO) and eyes-closed (EC) conditions at a sampling rate of 250 Hz.

Preprocessing was performed in MATLAB R2025b version 25.2 (MathWorks, Natick, MA, USA), using EEGLAB v2026.0.0 (Swartz Center for Computational Neuroscience, University of California San Diego, La Jolla, CA, USA). The pipeline included band-pass and line-noise filtering, removal of gross artifactual segments, average re-referencing, and independent component analysis to remove ocular, muscular, and other non-neural artifacts. No channels required removal or interpolation. Full preprocessing parameters and artifact-rejection procedures are provided in the Supplementary Methods.

### 2.4. Data Analysis

#### 2.4.1. Clinical Outcome Interpretation

Baseline and post-treatment values were reported together with their absolute change, calculated consistently as post-treatment minus baseline. Given the single-case design, no group-level inferential statistics were performed.

Individual change was examined using the Reliable Change Index (RCI), according to Jacobson and Truax [18], whenever numerical baseline and post-treatment observations and suitable psychometric parameters were available:

RCI = (X_post − X_pre)/S_diff,

where S_diff represents the standard error of the difference. When a published 95% minimal detectable change was available, S_diff was obtained by dividing the minimal detectable change by 1.96. RCI values were oriented so that positive values indicated change in the clinically favorable direction. Values of at least +1.96 were classified as reliable improvement. Values of −1.96 or lower were classified as reliable deterioration. Reliability parameters for the FIM were additionally informed by the quantitative review of Ottenbacher et al. [19]. Supplementary MoCA reliability estimates were informed by Bruijnen et al. [20]. Fox-Fuller et al. [21] was used as an indirect source for selected test–retest reliability estimates reported in Supplementary Table S1. These sources are detailed in Supplementary Table S1. Estimates derived from the same instrument and an appropriate clinical population were graded as directly applicable or compatible. Estimates based on different versions, administration modalities, languages, or clinical populations were graded as indirect. Proxy-based RCI calculations were retained only as exploratory sensitivity analyses and were not interpreted as confirmatory evidence. Complete psychometric parameters, applicability grades, calculations, and references are reported in Supplementary Table S1.

For the FAB, raw scores were converted to age- and education-adjusted scores for normative interpretation according to Appollonio et al. [10]. Age and education were unchanged between assessments. Demographic adjustment therefore did not affect the magnitude of the raw pre–post difference. Adjusted scores below 13.48 were considered below the normative cut-off [10]. An RCI was not calculated because an appropriate test–retest reliability coefficient was unavailable.

RCI findings were integrated with published clinical interpretability criteria. Changes in MoCA, FIM, and Modified Barthel Index scores were compared with available minimal clinically important difference or minimal detectable change thresholds [22,23,24,25,26]. Center of Cancellation values were compared with the pathological cut-offs of 0.081 for the Bells Cancellation Test and 0.083 for the Letter Cancellation Test [27]. HADS and Catherine Bergego Scale scores were interpreted according to their corresponding clinical ranges.

#### 2.4.2. Exploratory qEEG Analysis

Exploratory analyses examined resting-state spectral power and sensor-level functional connectivity separately for EO and EC conditions. Absolute power was estimated using Welch’s method. Power was then summarized within the following non-overlapping frequency bands: delta (1–4 Hz), theta (4–7.5 Hz), alpha (8–12 Hz), SMR (12–15 Hz), beta-1 (15–20 Hz), beta-2 (20–29 Hz), and low-gamma (29–45 Hz). Spectral values were summarized across the whole scalp, at Cz, and across predefined frontal, central, temporal, parietal, and occipital regions.

Sensor-level functional connectivity was estimated using the weighted Phase Lag Index (wPLI) in the theta, alpha, SMR, beta-1, beta-2, and low-gamma bands and summarized within and between the same scalp regions. Because this was a single-case exploratory analysis, no electrode-level or regional inferential statistics were performed.

A sensitivity analysis excluding high-amplitude epochs was conducted to assess the robustness of the spectral findings. Full spectral-estimation parameters, connectivity procedures, regional definitions, and sensitivity-analysis methods are reported in the Supplementary Methods. All electrophysiological analyses were descriptive and were not used to infer a treatment-specific neurophysiological mechanism.

## 3. Results

Results are presented according to the outcome domains described in the Methods. Baseline and post-treatment values, post-treatment minus baseline changes, RCI values, and clinical interpretability criteria are summarized in Table 3. To avoid duplication, the text reports the principal findings without restating all values included in the table. The multidimensional assessment included 94 variables. An RCI could be calculated for 80 of these, which had comparable numerical baseline and post-treatment observations together with suitable psychometric parameters. The remaining 14 outcomes were unsuitable for RCI calculation, either because an observation was absent, qualitative, categorical, or non-executable, or because an appropriate psychometric parameter was unavailable.

### 3.1. EEG-Neurofeedback Feasibility and Tolerability

The patient completed all 15 planned EEG-NF sessions, corresponding to an adherence rate of 100%. No session was interrupted or discontinued because of treatment intolerance. No adverse events were observed or reported during or immediately after training. Specifically, no clinically relevant fatigue, discomfort, headache, dizziness, nausea, agitation, or sustained worsening of neurological or cognitive symptoms were recorded. Feedback thresholds were adjusted by the clinician when required to maintain adequate engagement and reinforcement.

### 3.2. Cognitive and Affective Outcomes

The MoCA total score increased from 20/30 to 24/30. The change reached the published MCID but did not exceed the 5.1-point MDC95, and the RCI remained below the reliable-change threshold.

**Figure 3:**
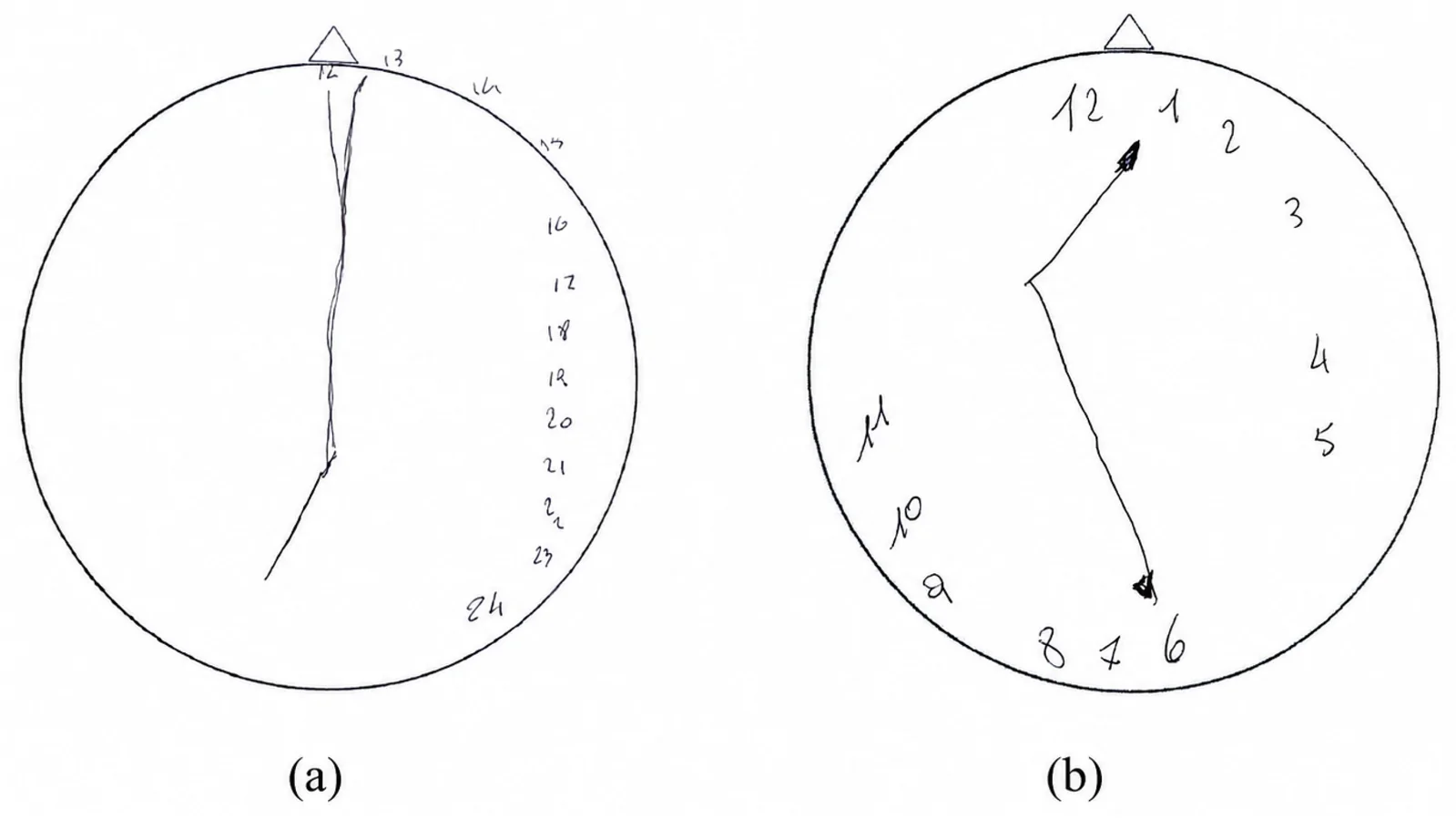
Clock Drawing Test performance before and after rehabilitation. The figure shows the patient’s drawings at baseline assessment (**a**) and post-treatment assessment (**b**). Qualitative comparison suggests improved spatial organization of the clock face and number placement after the multidisciplinary rehabilitation program.

The FAB raw score increased from 8 to 12. After adjustment for age and education according to Appollonio et al. [10], the adjusted score increased from 8.19 at baseline to 12.19 after treatment. Although the score improved by four points, both adjusted values remained below the normative cut-off of 13.48 [10]. An RCI was not calculated for the FAB because an appropriate test–retest reliability coefficient was unavailable. The Clock Drawing Test score increased from 5 to 9 (see Figure 3). Phonemic verbal fluency increased from 12 to 20 and semantic verbal fluency from 19 to 25; neither change exceeded the reliable-change threshold. Digit Span Forward remained unchanged at 4, whereas Digit Span Backward decreased from 3 to 2 without reliable deterioration. Stroop completion time decreased from 49 to 36 s, and the Stroop error-interference index changed from −3 to −1. Neither outcome met the confirmatory reliable-change threshold. On the Arrow Stroop Test, total responses increased from 53 to 103, and the number of errors decreased from 10 to 5, corresponding to a reduction in error percentage from 18.9% to 4.9%. Congruent-condition reaction time increased from 0.798 to 1.863 s, whereas incongruent-condition reaction time decreased from 1.118 to 0.935 s. HADS-Anxiety decreased from 4 to 3, whereas HADS-Depression remained unchanged at 1. Both scores remained within the non-clinical range.

### 3.3. Neglect-Specific and Ecological Outcomes

On the Bells Cancellation Test, detected targets increased from 5 to 17, and the Center of Cancellation decreased from 0.910 to 0.200. The post-treatment Center of Cancellation remained above the pathological cut-off of 0.081. On the Letter Cancellation Test, detected targets increased from 28 to 50, and the Center of Cancellation decreased from 0.522 to 0.070, crossing below the pathological cut-off of 0.083. Oxford Cognitive Screen spatial asymmetry decreased from 17 to 11.

On the Timed Neglect Test, left-sided target detection increased from 0 to 4, and the Neglect Index decreased from 1.44 to 0.26. Star-cancellation performance increased from 6 to 36, Diller letter-cancellation omissions decreased from 84 to 35, and article-reading performance increased from 3 to 9. Improvements were also recorded on the Baking Tray Task and during object search in the left extrapersonal space. The Catherine Bergego Scale decreased from 17 to 5, corresponding to a change from the moderate to the mild neglect range.

### 3.4. Neuromotor and Functional Outcomes

The FIM total score increased from 36 to 58, reaching the published post-stroke MCID and the reliable-change threshold. The FIM cognitive score increased from 19 to 32, exceeding both its MCID and reliable-change threshold. The FIM motor score increased from 17 to 26 but did not reach the published 17-point MCID or the reliable-change threshold.

The Modified Barthel Index increased from 5 to 21. The change exceeded both the adopted MCID equivalent and the published MDC95 of 15.4 points, and it met the reliable-change threshold.

The Timed Up and Go test was not executable at baseline. After treatment, the patient completed it in 19.72 s.

### 3.5. Exploratory qEEG Outcomes

All baseline and post-treatment EO and EC recordings were successfully processed and included in the exploratory analyses. Spectral changes were heterogeneous across recording conditions, frequency bands, and scalp regions. During EO, absolute delta and theta power increased broadly across regions, while alpha changes were spatially heterogeneous. SMR and higher-frequency activity generally increased, although the magnitude of change varied considerably across regions. During EC, delta, theta, and alpha power increased across regions. By contrast, SMR and beta-band changes were more region-dependent and included reductions over temporal regions. Some beta-2 and low-gamma measures showed particularly large percentage changes. These occurred against low baseline values and should therefore be interpreted cautiously. Complete regional values and topographic distributions are reported in Supplementary Table S2 and Supplementary Figure S1.

**Figure 4:**
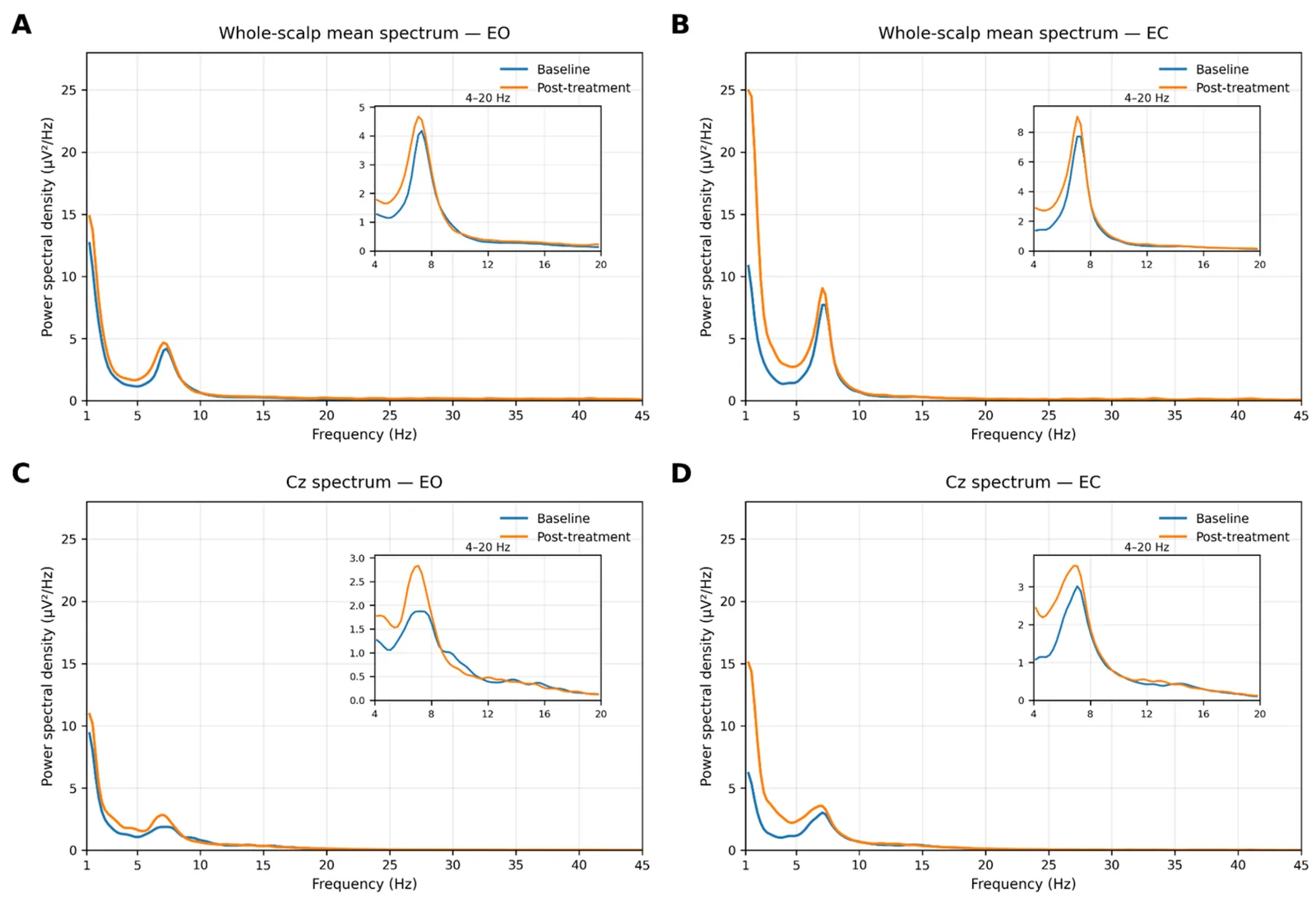
Frequency-resolved resting-state EEG power spectral density before and after rehabilitation: (**A**) whole-scalp mean spectrum during the eyes-open condition; (**B**) whole-scalp mean spectrum during the eyes-closed condition; (**C**) Cz spectrum during the eyes-open condition; and (**D**) Cz spectrum during the eyes-closed condition. Curves represent baseline and post-treatment power spectral density from 1 to 45 Hz. Insets provide an enlarged view of the 4–20 Hz range to improve visualization of alpha- and SMR-range activity. Inset y-axis limits were scaled independently within each panel and should not be compared across panels. Cz was the active EEG-neurofeedback training site. Spectra are presented descriptively as exploratory electrophysiological correlates of the patient’s clinical evolution.

The sensitivity analysis excluding high-amplitude epochs reproduced the principal spectral patterns observed in the primary analysis. Sensor-level wPLI also showed condition- and frequency-dependent changes rather than a uniform connectivity pattern. Notable findings included reduced frontal theta connectivity and increased frontal beta-2 connectivity during EO, together with increased beta-2 and low-gamma connectivity and reduced frontal–occipital alpha connectivity during EC. Complete regional connectivity values are provided in Supplementary Table S3 and Supplementary Figure S2. Frequency-resolved whole-scalp and Cz power spectral density curves are shown in Figure 4. All qEEG findings were descriptive and exploratory.

## 4. Discussion

This case report describes the clinical, neuropsychological, functional, and exploratory electrophysiological evolution of a 70-year-old patient with USN following a right thalamo-mesencephalic hemorrhagic stroke. The patient underwent an intensive multidisciplinary rehabilitation program including adjunctive EEG-NF. After treatment, convergent improvements were observed in neglect-specific performance, ecological neglect-related disability, selected executive-attentional measures, and functional independence. Exploratory qEEG analyses also identified condition- and region-dependent changes in absolute spectral power and sensor-level functional connectivity. The patient completed all 15 EEG-NF sessions without adverse events. However, neurofeedback was administered concurrently with physiotherapy, speech and language therapy, and conventional cognitive stimulation. None of the observed clinical or electrophysiological changes can therefore be attributed specifically to EEG-NF.

The present case is clinically relevant because it illustrates that USN is not exclusively associated with cortical injury. Neglect most commonly follows right fronto-parietal and temporo-parietal lesions. However, subcortical structures also contribute to spatial attention through distributed cortico-subcortical networks involved in arousal, attentional selection, executive control, and behavioral orienting [1,2,3,4,5]. In the present patient, the right thalamo-mesencephalic hemorrhage may therefore have disrupted thalamo-cortical and attentional network dynamics, contributing to the severe left-sided neglect observed at baseline. Nevertheless, the involvement of specific thalamic nuclei and their relationship with the clinical phenotype cannot be determined from the available structural imaging or from a single case [4].

The clinical outcome pattern indicated selective rather than generalized recovery. Neglect-specific measures showed the most consistent changes, including reduced spatial asymmetry, increased contralesional target detection, improved cancellation performance, and reduced ecological disability on the Catherine Bergego Scale. The Letter Cancellation Center of Cancellation crossed below the published pathological cut-off, whereas the Bells Cancellation Center of Cancellation improved substantially but remained within the pathological range [27]. These findings indicate a clinically meaningful reduction, but not a complete resolution, of spatial neglect. Cognitive changes were also selective. The MoCA improvement reached the published MCID but remained below the MDC95 [24,25]. FAB performance improved by four raw-score points; however, according to the Italian normative data of Appollonio et al. [10], the age- and education-adjusted score remained below the normative cut-off at post-treatment assessment. Functional gains were particularly evident in FIM total, FIM cognitive, and Modified Barthel Index scores, which met the corresponding clinical or reliable change criteria [22,23,26]. The dissociation between marked improvements in neglect-related and cognitive–functional outcomes and the more limited change in the FIM motor score is clinically noteworthy. Causal mediation cannot be inferred from this case. Nevertheless, improved attentional engagement, spatial exploration, and participation in rehabilitation may have supported greater functional independence despite persistent motor impairment. This is consistent with the recognized impact of spatial neglect on post-stroke functional outcomes [28]. Affective scores remained within the non-clinical range at both assessments, which limited the scope for detecting further improvement. The rationale for including EEG-NF was its potential to support self-regulation, attentional readiness, and sensorimotor engagement within the broader rehabilitation program. The present protocol differed substantially from the neglect-specific EEG-NF approaches reported previously. Ros et al. [6] used repeated P4-based alpha downregulation across six daily sessions, whereas Saj et al. [7] applied the same general posterior-alpha rationale in a single 30 min session during the acute post-stroke phase. By contrast, the present protocol used Cz as the active site, with reinforcement of SMR activity (12–15 Hz) and concurrent inhibition of theta (4–7.5 Hz) and high-beta (22–26 Hz) activity. A prospectively registered pragmatic open randomized trial specifically addressing post-stroke neglect (ISRCTN85291842) adopted a partially similar frequency-contingency approach. That protocol rewarded either SMR (12–15 Hz) or beta-1 (15–18 Hz) while inhibiting theta (4–7 Hz) and high-beta (22–30 Hz) activity across 30 planned sessions [29]. Importantly, however, that trial required right-hemisphere cortical involvement as an inclusion criterion, and the registry record does not provide study results [29]. The rt-fMRI neurofeedback study by Robineau et al. [9] investigated trained regulation of right visual cortex activity and provides additional proof of principle for neurofeedback in neglect. However, it relies on a different neurophysiological signal and target and is therefore not directly comparable with the present EEG-NF protocol.

Importantly, in the literature reviewed for the present report, we did not identify an EEG-NF study specifically designed to investigate thalamic or other subcortical neglect as a lesion-defined subgroup, nor a study stratifying EEG-NF outcomes according to lesion location [6,7,29]. Thus, the present case provides clinical documentation of EEG-NF embedded within multidisciplinary rehabilitation in a patient with a right thalamo-mesencephalic hemorrhagic lesion, but it should not be interpreted as evidence of lesion-specific efficacy. Whether patients with thalamic or other subcortical lesions respond differently from those with cortical lesions remains an open question. Existing studies differ markedly in post-stroke phase, neurophysiological target, training dose, and rehabilitation context [6,7,8,9,29]. At the time of writing, a targeted search of ClinicalTrials.gov and ISRCTN did not identify an actively recruiting trial specifically evaluating EEG-NF as a treatment for post-stroke spatial neglect; the dedicated EEG-NF neglect study ISRCTN85291842 is listed as completed [29].

Current research directions are increasingly extending beyond single-frequency neurofeedback toward adaptive EEG-based BCI, VR-integrated closed-loop systems, and network-informed rehabilitation targets [2,30,31]. Recent work on VR–BCI integration emphasizes individualized, ecologically valid, and adaptive rehabilitation environments, although clinical validation in the USN remains preliminary [30]. In parallel, Haddadshargh et al. [31] identified neglect-related EEG connectivity differences involving frontal and right parieto-occipital regions, particularly in theta and beta bands, with additional task-related differences in the gamma range, and proposed these network alterations as potential targets for future connectivity-based rehabilitation [31]. These developments remain exploratory and do not establish the efficacy of EEG-NF for USN; key priorities include appropriately controlled and lesion-stratified studies, repeated electrophysiological measurements, standardized neglect outcomes, and longer-term assessment of functional transfer [8,30,31]. The absolute-power analyses did not reveal a simple normalization of a single frequency band or a uniform neurofeedback-like effect. During the EO condition, delta, theta, SMR, beta-1, beta-2, and low-gamma power generally increased, whereas alpha changes differed across regions. During the EC condition, delta, theta, and alpha power increased across regions, while SMR and higher-frequency changes were spatially heterogeneous, including reductions over the temporal region. The particularly large percentage changes observed for some beta-2 and low-gamma measures should be considered alongside their absolute values because several corresponding baseline values were small. Overall, the findings are more consistent with a broad and condition-dependent redistribution of resting-state spectral activity than with selective modulation of the trained bands. One possible interpretation is that this distributed pattern reflects broader state-dependent changes in cortical activation, arousal, or vigilance rather than a narrowly frequency-specific modulation of the trained 12–15 Hz band. However, this interpretation remains hypothesis-generating. Resting-state EEG was acquired only before and after the rehabilitation period. The present design therefore cannot determine whether these changes were related to EEG-NF, spontaneous recovery, concurrent rehabilitation, or differences in physiological state between recording sessions.

The sensitivity analysis indicated that the principal spectral patterns were not driven by a small number of high-amplitude epochs. Nevertheless, resting-state EEG remains influenced by vigilance, fatigue, arousal, and other state-dependent factors that may vary across assessments in post-stroke patients. The spectral findings should therefore be regarded as exploratory correlates of the patient’s evolution rather than as biomarkers of treatment efficacy. This cautious interpretation is consistent with previous observations that unilateral thalamic and striato-thalamic hemorrhages may be accompanied by bilateral EEG abnormalities, reflecting disruption of distributed thalamo-cortical and cortico-thalamic networks [32].

The wPLI findings were similarly heterogeneous. During EO recording, theta connectivity decreased within frontal regions and between temporal and occipital regions, whereas beta-2 within-frontal connectivity increased. During EC recording, alpha frontal–occipital connectivity decreased, while beta-2 within-frontal and low-gamma central–temporal connectivity increased. The divergence between absolute band power and phase-based connectivity indicates that local oscillatory amplitude and large-scale synchronization did not necessarily change in parallel. The design was a single case, the electrode density was limited, and the analyses were performed at the sensor level. These findings therefore cannot be interpreted as evidence of a specific reorganization mechanism or of a causal pathway between EEG-NF and clinical recovery. From an integrated rehabilitation perspective, EEG-NF may have provided a framework for practicing cortical self-regulation and attentional readiness. In parallel, cognitive stimulation reinforced visuospatial exploration and compensatory strategies, while physiotherapy provided repeated sensorimotor input through posture, balance, weight-bearing, and gait-related practice [1,2,8]. The observed clinical evolution may therefore reflect interactions among complementary top-down and bottom-up rehabilitation components delivered during an intensive post-acute recovery period, rather than the isolated effect of any single intervention.

### Limitations

Several limitations should be explicitly acknowledged. First, this is an uncontrolled single-case report and therefore does not permit causal inference regarding treatment efficacy. Second, EEG-NF was delivered concurrently with physiotherapy, speech and language therapy, and conventional cognitive stimulation; consequently, the specific contribution of EEG-NF cannot be separated from that of the other rehabilitation components. Third, the patient was assessed during the post-acute recovery period. Spontaneous neurological recovery, repeated-testing effects, changes in arousal or engagement, and the cumulative intensity of rehabilitation may all have contributed to the observed clinical changes [33,34]. Fourth, the findings have limited generalizability because they derive from a single patient with a specific right thalamo-mesencephalic hemorrhagic lesion, clinical profile, and individualized rehabilitation program. Fifth, no long-term follow-up was available, preventing assessment of the durability of the observed clinical and electrophysiological changes. EEG-NF tolerability was also assessed pragmatically through clinical monitoring rather than with a standardized adverse-event instrument. Sixth, several exploratory RCI calculations relied on indirect or proxy psychometric parameters; these estimates were therefore treated as sensitivity analyses rather than confirmatory evidence [18]. Seventh, the qEEG analyses were exploratory and based on only two assessment time points. This precluded characterization of within-treatment electrophysiological trajectories and formal EEG–behavior association analyses. In addition, the 19-channel montage and sensor-level wPLI approach did not permit reliable source-level localization, while higher-frequency measures remain particularly sensitive to low baseline values and residual physiological noise. Accordingly, the electrophysiological findings should be regarded as descriptive and hypothesis-generating rather than as evidence of a treatment-specific neurophysiological mechanism. Future hypothesis-driven studies could strengthen causal inference by adopting single-case experimental designs with repeated measurements across baseline and intervention phases. In particular, a multiple-baseline design with staggered introduction of EEG-NF across participants, behaviors, or outcome domains could help distinguish changes temporally associated with the neurofeedback component from spontaneous recovery, repeated testing, and the effects of concurrent rehabilitation. Such designs also remain feasible in clinically heterogeneous neurorehabilitation populations [35] and would complement subsequent controlled group studies rather than replace them.

## 5. Conclusions

This case documents the feasibility of integrating 15 sessions of Cz-based EEG-NF into an intensive multidisciplinary inpatient rehabilitation program for a patient with USN following right thalamo-mesencephalic hemorrhagic stroke. Treatment was completed with full adherence and without adverse events. Clinically meaningful improvements were observed in neglect-related performance, ecological disability, and functional independence, alongside heterogeneous changes in resting-state spectral power and sensor-level connectivity.

Because of the uncontrolled single-case design, concurrent rehabilitation interventions, and absence of follow-up, these findings do not establish a specific therapeutic or neurophysiological effect of EEG-NF. Controlled longitudinal studies are required to determine whether EEG-NF provides benefits beyond spontaneous recovery and multidisciplinary standard care. Such studies should use standardized neglect outcomes, predefined EEG measures, appropriate comparison conditions, and longer-term assessments.

## Figures and Tables

**Table 1:** Multidisciplinary rehabilitation program.

Rehabilitation Component	Main Objectives	Frequency	Session Duration	Number of Sessions	Notes
**Physiotherapy**	Improvement of postural control, motor coordination, balance, endurance, and functional autonomy; recovery of left hemiplegia with focus on trunk control, weight-bearing symmetry, and gait	5 days/week	30 min.	15 sessions	Three structured phases: (1) bed positioning and left hemibody mobilization; (2) upright positioning with electric standing device and active-assisted upper limb exercises; (3) progressive gait training with gradual reduction in assistance.
**Speech, language, and swallowing therapy**	Management of dysphagia and swallowing functions; progression to soft solid diet; support of autonomous feeding and communication	5 days/week	30 min.	15 sessions	Electrical stimulation (VitalStim); C-TAR techniques; caregiver counseling for safe feeding and generalization to daily contexts
**Conventional cognitive stimulation**	Visual exploration, sustained attention, awareness of the contralesional hemispace, and compensatory strategies for USN	5 days/week (USN training); 2 days/week (Khymeia); 2 days/week (ecological group training); 1–2 days/week (paper-and-pencil tasks)	30 min.	15 sessions	USN training, Khymeia-based exercises, ecological group training, and paper-and-pencil tasks including complex-scene description tasks depicting real-life situations
**EEG-NF**	Cortical self-regulation, attentional stability, and modulation of EEG activity (enhancement of 12–15 Hz; inhibition of 4–7.5 Hz and 22–26 Hz bands) to support attentional control and visuospatial processing	5 days/week for 3 weeks	35 min.	15 sessions	Individualized protocol; active electrode at Cz (10–20 system); real-time visual and auditory feedback via gamified interface; clinician-adjusted threshold; monitoring of cognitive load and adverse events

**Table 2:** EEG-neurofeedback protocol parameters.

Parameter	Description
Device/software	ProComp5 system with BioGraph Infiniti software version 6.9.0.0 (Thought Technology, Montreal, QC, Canada)
Electrode placement	International 10–20 system; active electrode at Cz
Reference/ground	Reference: left ear; Ground: right ear
Reward band	12–15 Hz
Inhibitor bands	4–7.5 Hz and 22–26 Hz
Feedback modality	Real-time visual and auditory feedback via gamified interface
Session duration	35 min
Number of sessions	15 sessions (5 days/week for 3 weeks)
Threshold adjustment	Adaptively adjusted by the clinician during each session
Artifact monitoring	Sessions monitored for artifacts (e.g., movement, noise) and signal quality (impedance < 5 kΩ)
Adverse events	Monitored for fatigue, discomfort, reduced engagement, and adverse events

**Table 3:** Main clinical, neuropsychological, neglect-specific, affective, and functional outcomes.

Outcome	Baseline	Post-Treatment	Change	RCI	Clinical Interpretation
MoCA total	20	24	+4	1.54	MCID met; MDC95 not met
Frontal Assessment Battery	8	12	+4	N/A †	Adjusted score 8.19 → 12.19; below normative cut-off at both assessments
Clock Drawing Test	5	9	+4	Exploratory *	-
Digit Span Forward	4	4	+0	0.00	-
Digit Span Backward	3	2	−1	−0.48	-
Phonemic verbal fluency	12	20	+8	0.92	-
Semantic verbal fluency	19	25	+6	1.19	-
Stroop error-interference index	−3	−1	+2	Exploratory *	-
Stroop completion time (s)	49	36	−13	Exploratory *	-
OCS spatial asymmetry	17	11	−6	3.85	-
Bells Cancellation—Center of Cancellation	0.910	0.200	−0.710	Exploratory *	Improved but remained above pathological cut-off
Bells Cancellation—targets found	5	17	+12	Exploratory *	-
Letter Cancellation—Center of Cancellation	0.522	0.070	−0.452	Exploratory *	Post-treatment value below pathological cut-off
Letter Cancellation—targets found	28	50	+22	Exploratory *	-
TNT—left-sided target detection	0	4	+4	Exploratory *	-
TNT—Neglect Index	1.440	0.260	−1.180	Exploratory *	-
Baking Tray Task—mean deviation	−350	43	+393	Exploratory *	-
Star cancellation	6	36	+30	Exploratory *	-
Diller letter cancellation—total omissions	84	35	−49	Exploratory *	-
Article reading	3	9	+6	Exploratory *	-
Catherine Bergego Scale	17	5	−12	Exploratory *	Moderate → mild neglect
HADS—Anxiety	4	3	−1	0.43	Non-clinical at both assessments
HADS—Depression	1	1	+0	0.00	Non-clinical at both assessments
FIM total	36	58	+22	2.85	MCID met
FIM motor	17	26	+9	1.58	MCID not met
FIM cognitive	19	32	+13	4.84	MCID met
Modified Barthel Index	5	21	+16	2.04	MCID equivalent and MDC95 met
Timed Up and Go (s)	Not executable	19.720	N/A	N/A	Became executable

Note: Δ = post-treatment minus baseline; RCI = Reliable Change Index; MCID = minimal clinically important difference; MDC95 = 95% minimal detectable change; OCS = Oxford Cognitive Screen; FIM = Functional Independence Measure; HADS = Hospital Anxiety and Depression Scale; TNT = Timed Neglect Test. RCI values are reported in the main table only when based on direct or compatible psychometric evidence. Grade-C proxy-based RCI estimates are reported exclusively in Supplementary Table S1 and are interpreted as exploratory sensitivity analyses. Positively oriented RCI values indicate change in the clinically favorable direction. For the FAB, age- and education-adjusted scores were calculated according to Appollonio et al. [10]; both adjusted scores remained below the normative cut-off. † RCI was not calculated because an appropriate test–retest reliability coefficient was unavailable. * Proxy-based RCI estimate retained only as an exploratory sensitivity analysis and not interpreted as confirmatory evidence.

## Data Availability

The clinical and neurophysiological data presented in this study are available from the corresponding author upon request, subject to privacy and ethical restrictions. The custom MATLAB scripts used for spectral and connectivity analyses are also available from the corresponding author upon request. The data are not publicly available because they relate to a single clinical case.

## Supplementary Materials

The following supporting information can be downloaded at https://www.mdpi.com/article/10.3390/neurolint18090167/s1, Table S1: Complete individual reliable-change analysis across clinical, neuropsychological, affective, neglect-specific, motor, and functional outcomes; Table S2: Summary of regional absolute qEEG power changes using non-overlapping frequency bands; Table S3: Selected regional sensor-level wPLI connectivity changes; Figure S1: Complete absolute-power topographic maps for eyes-open and eyes-closed conditions across the predefined non-overlapping frequency bands; Figure S2: Regional sensor-level wPLI connectivity heatmaps for eyes-open and eyes-closed conditions across the analyzed frequency bands. The psychometric references used for Supplementary Table S1 are included in the main reference list [10,11,12,14,15,16,17,18,19,20,21,22,23,24,25,26,27].

## Abbreviations

qEEG	Quantitative Electroencephalography
wPLI	Weighted Phase Lag Index
EEG-NF	Electroencephalography-Based Neurofeedback
SMR	Sensorimotor Rhythm
RCI	Reliable Change Index
MCID	Minimal Clinically Important Difference
MDC95	95% Minimal Detectable Change
FIM	Functional Independence Measure
MBI	Modified Barthel Index
HADS	Hospital Anxiety and Depression Scale
CBS	Catherine Bergego Scale
TUG	Timed Up and Go
