# Supplementary material for: Integrated Neurorehabilitation Including EEG-Neurofeedback for Unilateral Spatial Neglect After Right Thalamo-Mesencephalic Intracerebral Hemorrhage: A Case Report
Source: Neurol Int. 2026 Aug 27;18(9):167. doi: 10.3390/neurolint18090167 (PMC13610569; doi:10.3390/neurolint18090167)
Supplement: Supplementary file 1 [file neurolint-18-00167-s001.zip › neurolint-4487183-supplementary.pdf]

Supplementary Materials S1

Integrated Neurorehabilitation Including EEG-Neurofeedback for Unilateral Spatial Neglect After Right Thalamo-Mesencephalic Intracerebral Hemorrhage: A Case Report

Supplementary Methods. Detailed EEG Preprocessing and qEEG Analysis

Resting-state EEG was recorded using a Mitsar-201 EEG system and 19 Ag/AgCl electrodes positioned according to the international 10–20 system. Recordings were obtained under both eyes-open (EO) and eyes-closed (EC) conditions for approximately 6.3 min per condition, at a sampling rate of 250 Hz. Electrode impedance was maintained below 5 kΩ whenever possible. EEG preprocessing was performed in MATLAB R2025b, version 25.2 (MathWorks, Natick, MA, USA), using EEGLAB v2026.0.0. Only the 19 standard EEG channels were retained, and standard electrode locations were assigned using the EEGLAB standard_1005 coordinate file. Continuous data were band-pass filtered between 1 and 80 Hz, and 50-Hz line noise was attenuated using a 48–52 Hz notch filter.

After visual identification and removal of gross artefactual segments, data were re-referenced to the average reference. Extended Infomax independent component analysis was then performed with rank correction. Components were classified using ICLabel and visually reviewed before rejection. Components were removed only when their topography, time course, and classification were consistent with ocular, muscular, or other non-neural artefactual activity. No channels required removal or interpolation. Following component rejection, the data were again re-referenced to the average reference and retained for spectral and connectivity analyses.

For spectral analysis, each continuous EEG channel was linearly detrended before spectral estimation. Power spectral density was estimated using Welch's method with 2-s symmetric Hann windows, 50% overlap, and a 1024-point fast Fourier transform. Within each segment, the mean was removed before windowing, and the resulting one-sided power spectral density estimates were averaged across segments. Absolute power was selected as the primary spectral metric.

Absolute band power was calculated by trapezoidal integration of the power spectral density within the following non-overlapping frequency bands: delta (1–4 Hz), theta (4–7.5 Hz), alpha (8–12 Hz), sensorimotor rhythm (SMR; 12–15 Hz), beta-1 (15–20 Hz), beta-2 (20–29 Hz), and low-gamma (29–45 Hz). SMR corresponded to the EEG-NF reward band, whereas beta-2 included the 22–26 Hz inhibitory range used during training. For each band and recording condition, absolute change was calculated as post-treatment minus baseline, and percentage change was calculated relative to the baseline value. EO and EC conditions were analyzed separately. Frequency-resolved power spectral density was summarized across all 19 electrodes and separately at Cz, the active EEG-NF training site.

Band-power values were additionally summarized across five predefined scalp regions: frontal (Fp1, Fp2, F7, F3, Fz, F4, and F8), central (C3, Cz, and C4), temporal (T7, T8, P7, and P8), parietal (P3, Pz, and P4), and occipital (O1 and O2). Because electrodes within a single participant cannot be considered statistically independent observations, no inferential statistics were performed on electrode-level or regional values. Analyses focused on the direction, magnitude, and spatial distribution of post-treatment changes.

A sensitivity analysis was performed to determine whether spectral findings were driven by residual high-amplitude segments. Each final recording was divided into non-overlapping 2-s epochs. For each recording, a robust z-score was calculated from epoch-level log-transformed broadband power as the difference between each value and the median, divided by 1.4826 times the median absolute deviation. Epochs with a robust z-score greater than 3.5 were classified as high-amplitude outliers and excluded. Spectral estimates were then recalculated using the retained epochs and summarized using median values.

Sensor-level functional connectivity was estimated using the weighted Phase Lag Index (wPLI), selected to reduce the influence of zero-lag synchronization potentially associated with volume conduction. Connectivity estimation used 2-s Hann-windowed segments, 50% overlap, and a 1024-point fast Fourier transform. Each channel was mean-centered within each segment before windowing. For each channel pair and frequency band, the imaginary component of the cross-spectral density was pooled across segments and frequency bins within the band. wPLI was calculated as the absolute mean of the imaginary cross-spectrum divided by the mean of its absolute value.

Regional connectivity values were obtained by averaging pairwise wPLI estimates across all channel pairs within and between the five predefined scalp regions. Connectivity was summarized separately for EO and EC conditions in the theta, alpha, SMR, beta-1, beta-2, and low-gamma bands. Delta-band connectivity was not emphasized because of its greater sensitivity to slow artifacts, vigilance fluctuations, and residual non-stationary activity.

Tables S1–S3 and Figures S1–S2

Supplementary Table S1. Complete individual reliable-change analysis across clinical, neuropsychological, affective, neglect-specific, motor, and functional outcomes.

Measure	Baseline	Post	Δ	Method	SD/MDC/SD diff	r/ICC	Sdiff	Oriented RCI	Interpretation	Grade
MoCA total	20	24	+4	MDC95	5.100	N/A	2.602	1.54	No reliable change	A
MoCA - Executive	0	3	+3	SD + r	0.630	0.440	0.667	4.50	Reliable improvement	B
MoCA - Naming	3	3	+0	SD + r	0.360	0	0.509	0	No exploratory reliable change	C
MoCA - Memory	3	4	+1	SD + r	1.040	0.300	1.231	0.81	No reliable change	B
MoCA - Attention	4	4	+0	SD + r	0.690	0.490	0.697	0	No reliable change	B
MoCA - Language	2	2	+0	SD + r	0.680	0.180	0.871	0	No reliable change	B
MoCA - Abstraction	2	2	+0	SD + r	0.550	0.310	0.646	0	No reliable change	B
MoCA - Orientation	6	6	+0	SD + r	0.260	0.540	0.249	0	No reliable change	B
Frontal Assessment Battery	8	12	+4	Normative classification	N/A	N/A	N/A	N/A	Adjusted score 8.19 → 12.19; both values remained below the normative cut-off of 13.48; RCI not calculated	Normative
Clock Drawing Test	5	9	+4	SD + r	2.900	0.774	1.950	2.05	Exploratory improvement	C
OCS - Naming	4	4	+0	SD + r	1	0.331	1.157	0	No exploratory reliable change	C
OCS - Semantics	3	3	+0	SD + r	0.750	0.776	0.502	0	No exploratory reliable change	C
OCS - Orientation	3	4	+1	SD + r	1	0.490	1.010	0.99	No exploratory reliable change	C
OCS - Visual field	4	4	+0	SD + r	1	0.628	0.863	0	No exploratory reliable change	C
OCS - Sentence reading	15	15	+0	SD + r	3.750	0.735	2.730	0	No exploratory reliable change	C
OCS - Number writing	3	3	+0	SD + r	0.750	0.605	0.667	0	No exploratory reliable change	C
OCS - Calculation	3	4	+1	SD + r	1	0.462	1.037	0.96	No exploratory reliable change	C
OCS - Hearts total	22	22	+0	SD + r	12.500	0.733	9.134	0	No exploratory reliable change	C
OCS - Object asymmetry	3	3	+0	SD + r	0.632	0.709	0.482	0	No reliable change	B
OCS spatial asymmetry	17	11	-6	SD + r	1.518	0.472	1.560	3.85	Reliable improvement	B
OCS - Gesture imitation	8	12	+4	SD + r	3	0.575	2.766	1.45	No exploratory reliable change	C
OCS - Free recall	0	3	+3	SD + r	1	0.767	0.683	4.39	Exploratory improvement	C
OCS - Verbal-memory recognition	2	4	+2	SD + r	1	0.640	0.849	2.36	Exploratory improvement	C

Measure	Baseline	Post	Δ	Method	SD/MDC/SD diff	r/ICC	Sdiff	Oriented RCI	Interpretation	Grade
OCS - Episodic-memory recognition	4	4	+0	SD + r	1	0.601	0.893	0	No exploratory reliable change	C
OCS executive task - baseline	Not executable	10	N/A	NC	N/A	N/A	N/A	N/A	Not calculable	NC
OCS executive task - mixed condition	Not executable	0	N/A	NC	N/A	N/A	N/A	N/A	Not calculable	NC
OCS executive task - shifting index	Not executable	10	N/A	NC	N/A	N/A	N/A	N/A	Not calculable	NC
Digit Span Forward	4	4	+0	SD of change	2.110	0.610	2.110	0	No reliable change	B
Digit Span Backward	3	2	-1	SD of change	2.070	0.660	2.070	-0.48	No reliable change	B
Phonemic verbal fluency	12	20	+8	SD of change	8.670	0.760	8.670	0.92	No reliable change	B
Semantic verbal fluency	19	25	+6	SD of change	5.060	0.520	5.060	1.19	No reliable change	B
Symbol Digit Modalities Test	Not executable	4	N/A	NC	N/A	N/A	N/A	N/A	Not calculable	NC
Stroop error-interference index	-3	-1	+2	SD + r	1.030	0.500	1.030	1.94	No exploratory reliable change	C
Stroop completion time (s)	49	36	-13	SD + r	8.380	0.600	7.495	1.73	No exploratory reliable change	C
Bells Cancellation - Center of Cancellation	0.910	0.200	-0.710	SD + r	0.031	0.830	0.018	39.79	Exploratory improvement	C
Bells Cancellation - targets found	5	17	+12	SD + r	8.750	0.830	5.102	2.35	Exploratory improvement	C
Letter Cancellation - Center of Cancellation	0.522	0.070	-0.452	SD + r	0.031	0.830	0.018	25.33	Exploratory improvement	C
Letter Cancellation - targets found	28	50	+22	SD + r	15	0.830	8.746	2.52	Exploratory improvement	C
TNT - left-sided target detection	0	4	+4	SD + r	1.500	0.830	0.875	4.57	Exploratory improvement	C
TNT - central target detection	4	6	+2	SD + r	1.500	0.830	0.875	2.29	Exploratory improvement	C
TNT - right-sided target detection	5	6	+1	SD + r	1.500	0.830	0.875	1.14	No exploratory reliable change	C
TNT - left reaction time (s)	5	3.720	-1.280	SD + r	1	0.800	0.632	2.02	Exploratory improvement	C
TNT - central reaction time (s)	3.800	3.360	-0.440	SD + r	1	0.800	0.632	0.70	No exploratory reliable change	C
TNT - right reaction time (s)	3.560	3.470	-0.090	SD + r	1	0.800	0.632	0.14	No exploratory reliable change	C
TNT - Neglect Index	1.440	0.260	-1.180	SD + r	0.500	0.800	0.316	3.73	Exploratory improvement	C
Baking Tray Task - left items	0	8	+8	SD + r	3	0.830	1.749	4.57	Exploratory improvement	C
Baking Tray Task - right items	12	4	-8	SD + r	3	0.830	1.749	4.57	Exploratory improvement	C

Measure	Baseline	Post	Δ	Method	SD/MDC/SD diff	r/ICC	Sdiff	Oriented RCI	Interpretation	Grade
Baking Tray Task - mean deviation	-350	43	+393	SD + r	175	0.830	102.042	3.01	Exploratory improvement	C
Arrow Stroop - total responses	53	103	+50	SD + r	30	0.830	17.493	2.86	Exploratory improvement	C
Arrow Stroop - errors	10	5	-5	SD + r	5	0.830	2.915	1.71	No exploratory reliable change	C
Arrow Stroop - errors (%)	18.868	4.854	-14.014	SD + r	25	0.830	14.577	0.96	No exploratory reliable change	C
Arrow Stroop - congruent RT (s)	0.798	1.863	+1.065	SD + r	0.300	0.926	0.115	-9.23	Exploratory deterioration	C
Arrow Stroop - incongruent RT (s)	1.118	0.935	-0.183	SD + r	0.300	0.926	0.115	1.59	No exploratory reliable change	C
Arrow Stroop - RT difference (s)	0.320	-0.928	-1.248	SD + r	0.400	0.500	0.400	-1.52	No exploratory reliable change	C
Awareness - hemiparesis	2	3	+1	SD + r	0.750	0.830	0.437	2.29	Exploratory improvement	C
Awareness - somatosensory deficit	0	3	+3	SD + r	0.750	0.830	0.437	6.86	Exploratory improvement	C
Awareness - hemianopia	1	1	+0	SD + r	0.750	0.830	0.437	0	No exploratory reliable change	C
Awareness - neglect	0	3	+3	SD + r	0.750	0.830	0.437	6.86	Exploratory improvement	C
Awareness - dysarthria	0	1	+1	SD + r	0.750	0.830	0.437	2.29	Exploratory improvement	C
Awareness - language production	1	1	+0	SD + r	0.750	0.830	0.437	0	No exploratory reliable change	C
Awareness - language comprehension	1	1	+0	SD + r	0.750	0.830	0.437	0	No exploratory reliable change	C
HADS - Anxiety	4	3	-1	SD + r	4.200	0.850	2.300	0.43	No reliable change	B
HADS - Depression	1	1	+0	SD + r	3.700	0.840	2.093	0	No reliable change	B
Personal neglect - tactile double stimulation	14	15	+1	SD + r	3.750	0.830	2.187	0.46	No exploratory reliable change	C
Personal neglect - visual double stimulation	7	12	+5	SD + r	3.750	0.830	2.187	2.29	Exploratory improvement	C
Personal Neglect Scale	7	8	+1	SD + r	2.250	0.830	1.312	-0.76	No exploratory reliable change	C
Line cancellation	35	35	+0	SD + r	9	0.830	5.248	0	No exploratory reliable change	C
Letter cancellation (A4)	27	27	+0	SD + r	10	0.830	5.831	0	No exploratory reliable change	C
Star cancellation	6	36	+30	SD + r	13.500	0.830	7.872	3.81	Exploratory improvement	C
Figure copying	0	Apraxia	N/A	NC	N/A	N/A	N/A	N/A	Not calculable	NC
Line bisection	Missing	7	N/A	NC	N/A	N/A	N/A	N/A	Not calculable	NC
Spontaneous drawing	Missing	2	N/A	NC	N/A	N/A	N/A	N/A	Not calculable	NC
Diller letter cancellation - total omissions	84	35	-49	SD + r	26	0.830	15.160	3.23	Exploratory improvement	C
Diller letter cancellation - right omissions	32	5	-27	SD + r	13	0.830	7.580	3.56	Exploratory improvement	C

Measure	Baseline	Post	Δ	Method	SD/MDC/SD diff	r/ICC	Sdiff	Oriented RCI	Interpretation	Grade
Diller letter cancellation - left omissions	52	30	-22	SD + r	13	0.830	7.580	2.90	Exploratory improvement	C
Diller letter cancellation - lateral difference	-20	-25	-5	SD + r	26	0.830	15.160	-0.33	No exploratory reliable change	C
Article reading	3	9	+6	SD + r	2.250	0.830	1.312	4.57	Exploratory improvement	C
Sentence reading	6	6	+0	SD + r	1.500	0.830	0.875	0	No exploratory reliable change	C
Complex-scene description	3	3	+0	SD + r	0.750	0.830	0.437	0	No exploratory reliable change	C
Environment description	Missing	General symmetry	N/A	NC	N/A	N/A	N/A	N/A	Not calculable	NC
Tea ceremony task	3	1	-2	SD + r	0.750	0.830	0.437	4.57	Exploratory improvement	C
Card distribution task	3	1	-2	SD + r	0.750	0.830	0.437	4.57	Exploratory improvement	C
Object search - right	8	12	+4	SD + r	3	0.830	1.749	2.29	Exploratory improvement	C
Object search - left	0	6	+6	SD + r	3	0.830	1.749	3.43	Exploratory improvement	C
Motor neglect - folding paper	Not applicable	1	N/A	NC	N/A	N/A	N/A	N/A	Not calculable	NC
Motor neglect - tearing paper	Not applicable	0	N/A	NC	N/A	N/A	N/A	N/A	Not calculable	NC
Motor neglect - paper and ruler	Not applicable	0	N/A	NC	N/A	N/A	N/A	N/A	Not calculable	NC
Catherine Bergego Scale	17	5	-12	SD + r	7.500	0.950	2.372	5.06	Exploratory improvement	C
FIM total	36	58	+22	SD + r	24.400	0.950	7.716	2.85	Reliable improvement	B
FIM motor	17	26	+9	SD + r	18	0.950	5.692	1.58	No reliable change	B
FIM cognitive	19	32	+13	SD + r	8.500	0.950	2.688	4.84	Reliable improvement	B
Modified Barthel Index	5	21	+16	MDC95	15.400	N/A	7.857	2.04	Reliable improvement	A
Timed Up and Go - theoretical fall risk	Not executable	High	N/A	NC	N/A	N/A	N/A	N/A	Not calculable	NC
Timed Up and Go (s)	Not executable	19.720	N/A	NC	N/A	N/A	N/A	N/A	Not calculable	NC

RCI was calculated for outcomes with two comparable numerical observations and suitable psychometric parameters. Direct or compatible estimates (grades A–B) are separated from proxy-based sensitivity analyses (grade C). Grade-C estimates are exploratory and must not be interpreted as confirmatory evidence. Non-numerical, categorical, absent, or non-executable observations were classified as non-calculable.

Note. Δ = post-treatment minus baseline. The oriented RCI is positive when change occurs in the clinically favorable direction; for asymmetry measures, improvement was defined as movement toward zero. For grade-C analyses, standard deviations and/or reliability coefficients were derived from related measures, scale dispersion, or indirect populations. These estimates are retained only as exploratory sensitivity analyses. NC = not calculable. For the Frontal Assessment Battery, raw scores were adjusted for age and education using Appollonio et al. [10]; the adjusted score changed from 8.19 to 12.19, with both values remaining below the normative cut-off of 13.48. RCI was not calculated because an appropriate test–retest reliability coefficient was unavailable.

Supplementary Table S2. Summary of regional absolute qEEG power changes using non-overlapping frequency bands.

Condition	Band	Regional pattern	Lowest regional Δ (%)	Highest regional Δ (%)	Comment
EO	Delta	Increase across all regions	3.351 (Occipital)	46.921 (Central)	
EO	Theta	Increase across all regions	20.125 (Parietal)	66.544 (Central)	
EO	Alpha	Heterogeneous	-10.274 (Frontal)	17.277 (Occipital)	Frontal and parietal reductions; temporal and occipital increases; central approximately stable
EO	SMR	Increase across all regions	6.554 (Frontal)	68.522 (Occipital)	
EO	Beta-1	Increase across all regions	15.590 (Frontal)	95.582 (Occipital)	
EO	Beta-2	Increase across all regions	43.745 (Frontal)	264.402 (Central)	
EO	Low-gamma	Increase across all regions	72.162 (Frontal)	472.315 (Central)	Large percentage changes partly reflect low baseline values
EC	Delta	Increase across all regions	132.560 (Temporal)	167.325 (Frontal)	
EC	Theta	Increase across all regions	29.526 (Temporal)	74.987 (Central)	
EC	Alpha	Increase across all regions	3.583 (Frontal)	27.7 (Occipital)	
EC	SMR	Region-dependent	-19.305 (Temporal)	Positive in all non-temporal regions	
EC	Beta-1	Region-dependent	-33.975 (Temporal)	Positive in all non-temporal regions	
EC	Beta-2	Region-dependent	-35.186 (Temporal)	Positive in all non-temporal regions	
EC	Low-gamma	Increase across all regions	1.031 (Temporal)	368.4 (Central)	Large percentage changes partly reflect low baseline values

Absolute-power changes were calculated as post-treatment minus baseline. EO and EC conditions were analyzed separately. The table summarizes the direction and regional range of the observed percentage changes; relative-power, log-transformed power, and spectral-ratio results are not included.

Note. EO = eyes open; EC = eyes closed; SMR = sensorimotor rhythm. Frequency bands: delta 1–4 Hz, theta 4–7.5 Hz, alpha 8–12 Hz, SMR 12–15 Hz, beta-1 15–20 Hz, beta-2 20–29 Hz, and low-gamma 29–45 Hz. Large percentage changes in higher-frequency bands should be interpreted cautiously when baseline absolute-power values were low.

Supplementary Table S3. Selected regional sensor-level wPLI connectivity changes.

Condition	Band	Regional connection	Baseline wPLI	Post-treatment wPLI	ΔwPLI	Δ (%)
EC	Low-gamma	Central–Temporal	0.054	0.272	+0.219	+406.3
EC	Beta-2	Frontal–Frontal	0.04	0.203	+0.163	+409.2
EC	Low-gamma	Frontal–Temporal	0.06	0.205	+0.145	+242.6
EC	Low-gamma	Frontal–Frontal	0.099	0.241	+0.142	+142.7
EC	Low-gamma	Temporal–Parietal	0.061	0.202	+0.142	+233.8
EO	Theta	Frontal–Frontal	0.27	0.131	-0.139	-51.5
EC	Alpha	Frontal–Occipital	0.314	0.188	-0.126	-40.3
EO	Beta-2	Frontal–Frontal	0.07	0.195	+0.125	+179.7
EO	Theta	Temporal–Occipital	0.276	0.166	-0.110	-39.9

The table reports the regional connections highlighted in the main text together with additional large absolute changes available from the regional summary. Rows are ordered approximately by absolute ΔwPLI. Values are descriptive.

Note. ΔwPLI = post-treatment minus baseline. EO = eyes open; EC = eyes closed; wPLI = weighted Phase Lag Index. Percentage changes can be large when baseline wPLI values are low and should therefore be interpreted together with absolute ΔwPLI.

Supplementary Figure S1. Complete absolute-power topographic maps for eyes-open and eyes-closed conditions across the predefined non-overlapping frequency bands.

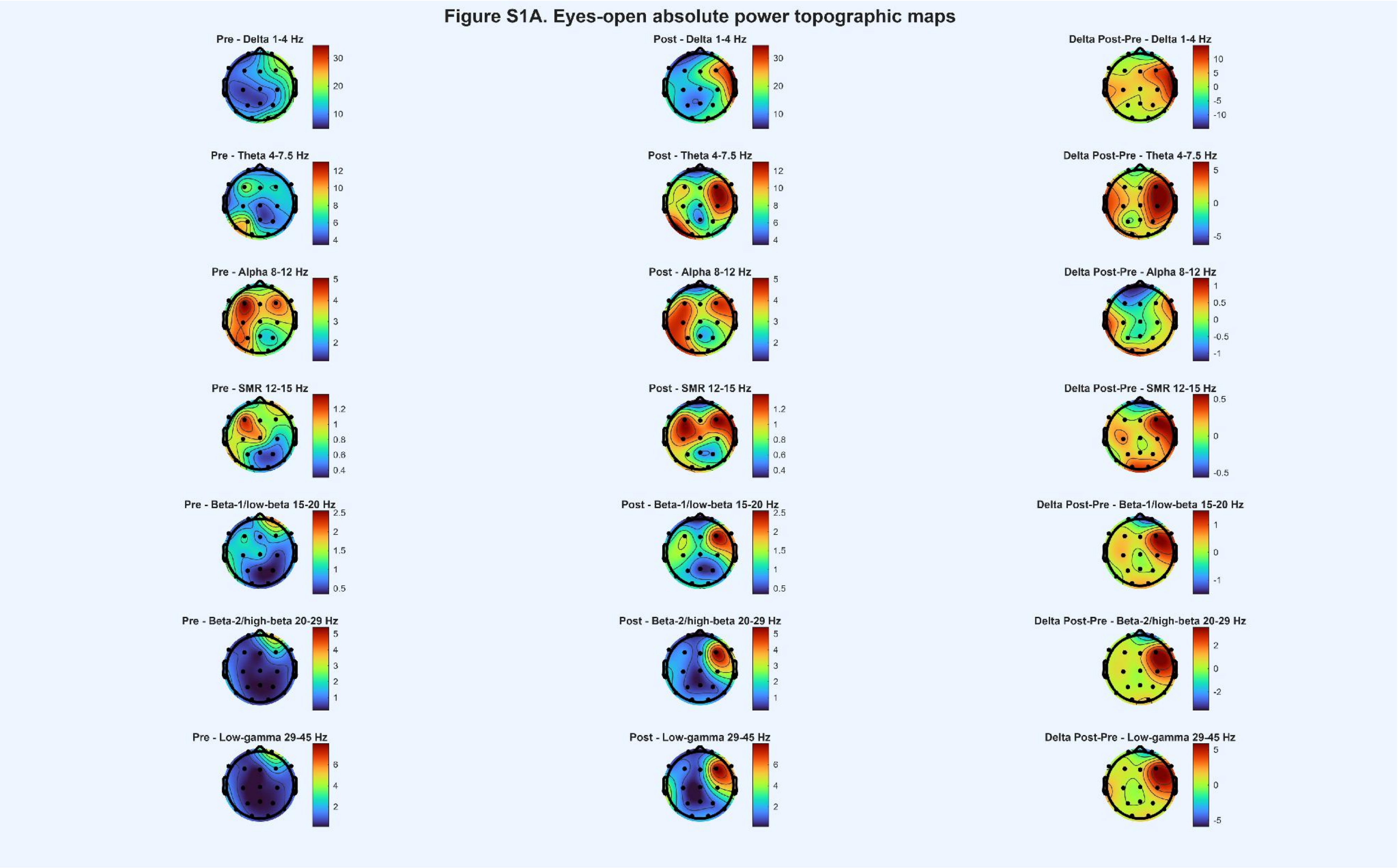

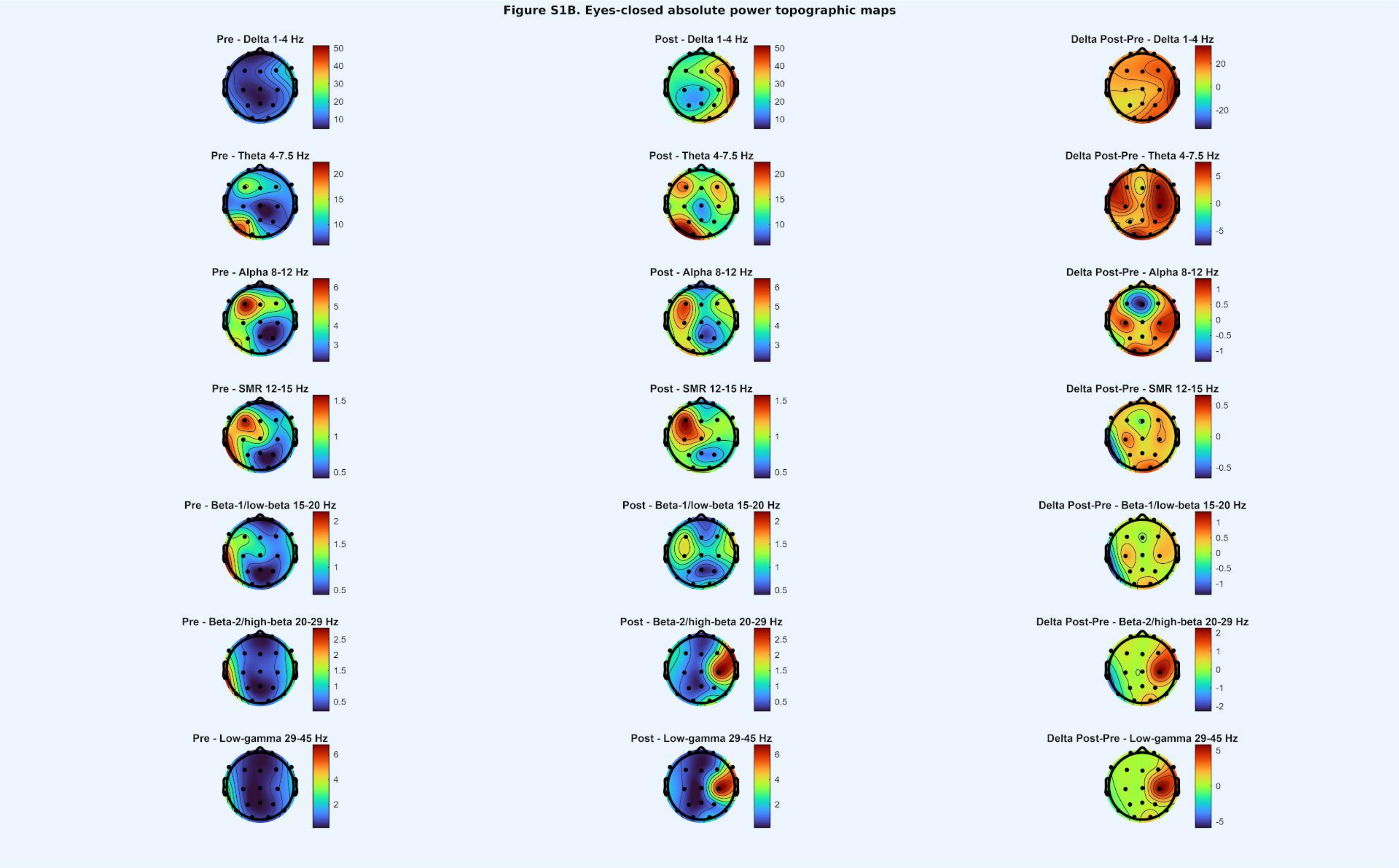

(A) Eyes-open condition; (B) eyes-closed condition. For each band, maps show baseline, post-treatment, and Δ values calculated as post-treatment minus baseline. Baseline and post-treatment maps share the same color scale within each band, whereas Δ maps use a symmetric scale centered on zero. Color scales were set separately for each band and should not be compared directly across bands. Maps are presented descriptively as exploratory electrophysiological correlates of clinical evolution.

Supplementary Figure S2. Regional sensor-level wPLI connectivity heatmaps for eyes-open and eyes-closed conditions across the analyzed frequency bands.

Figure S2A. Eyes-open sensor-level wPLI connectivity heatmaps

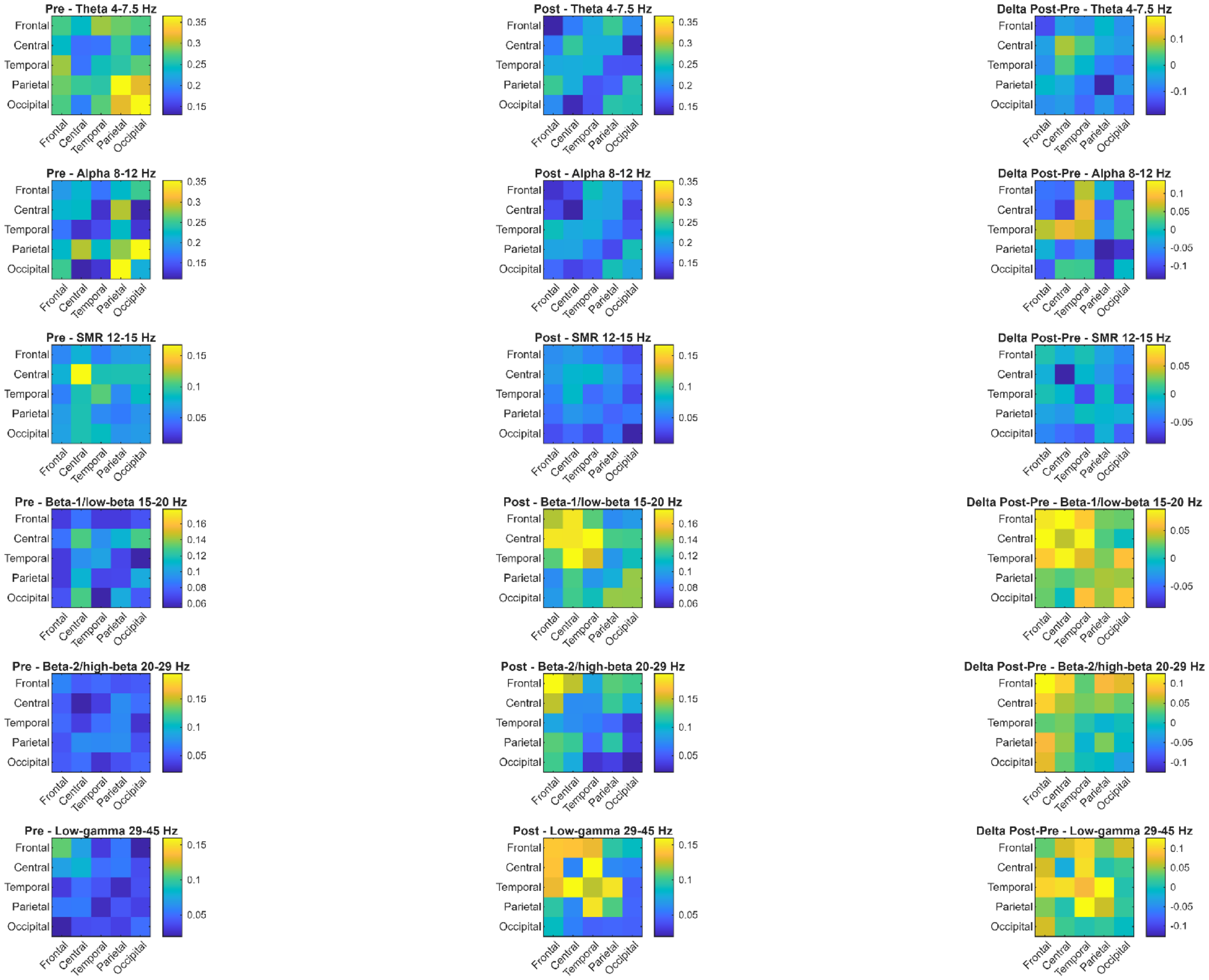

Figure S2B. Eyes-closed sensor-level wPLI connectivity heatmaps

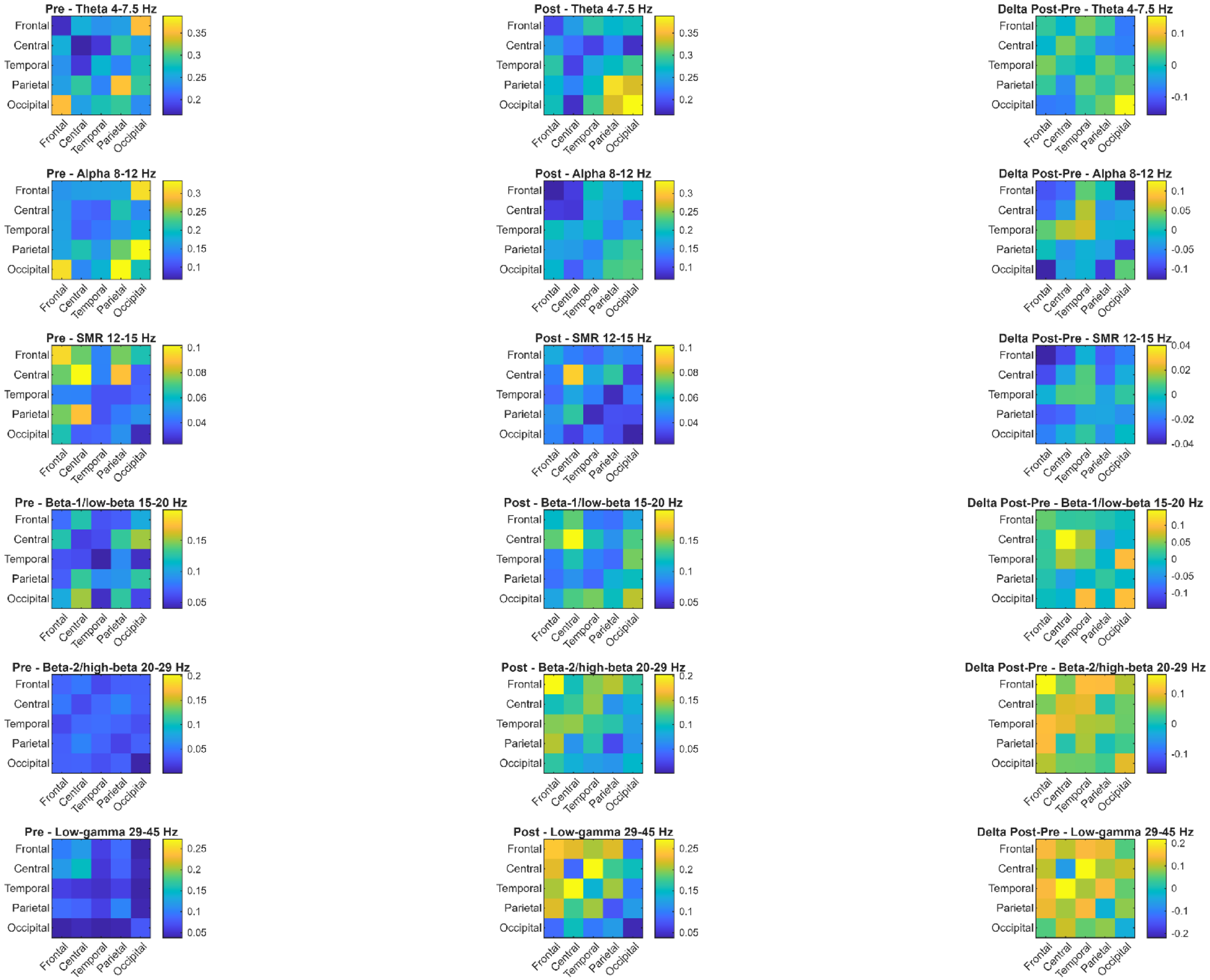

(A) Eyes-open condition; (B) eyes-closed condition. For each band, matrices show baseline, post-treatment, and ΔwPLI (post-treatment minus baseline) across frontal, central, temporal, parietal, and occipital regions. Baseline and post-treatment share the same color scale within each band; Δ uses a symmetric scale centered on zero. Scales differ across bands. Values are descriptive and not inferential.

References used for Supplementary Table S1

10. Appollonio, I.; Leone, M.; Isella, V.; Piamarta, F.; Consoli, T.; Villa, M.L.; Forapani, E.; Russo, A.; Nichelli, P. The Frontal Assessment Battery (FAB): Normative values in an Italian population sample. Neurol. Sci. 2005, 26, 108–116. https://doi.org/10.1007/s10072-005-0443-4
11. Aguilar-Navarro, S.G.; Mimenza-Alvarado, A.J.; Samudio-Cruz, M.A.; Hernández-Contreras, F.J.; Gutiérrez-Gutiérrez, L.A.; Ramírez-González, F.; Ávila-Funes, J.A. Validation of the Clock Drawing Test scoring method in older adults with neurocognitive disorder. Salud Ment. 2018, 41, 179–186. https://doi.org/10.17711/SM.0185-3325.2018.026.
12. Caffarra, P.; Vezzadini, G.; Dieci, F.; Zonato, F.; Venneri, A. A short version of the Stroop test: Normative data in an Italian population sample. Nuova Riv. Neurol. 2002, 12, 111–115.
14. Karlsson, J.; Hammarström, E.; Fogelkvist, M.; Lundqvist, L.O. Psychometric characteristics of the Hospital Anxiety and Depression Scale in stroke survivors of working age before and after inpatient rehabilitation. PLoS ONE 2024, 19, e0306754. https://doi.org/10.1371/journal.pone.0306754.
15. Demeyere, N.; Riddoch, M.J.; Slavkova, E.D.; Bickerton, W.L.; Humphreys, G.W. The Oxford Cognitive Screen: Validation of a stroke-specific short cognitive screening tool. Psychol. Assess. 2015, 27, 883–894. https://doi.org/10.1037/pas0000082.
16. Mancuso, M.; Varalta, V.; Sardella, L.; Capitani, D.; Zoccolotti, P.; Antonucci, G.; Italian OCS Group. Italian normative data for a stroke-specific cognitive screening tool: The Oxford Cognitive Screen. Neurol. Sci. 2016, 37, 1713–1721. https://doi.org/10.1007/s10072-016-2650-6.
17. Azouvi, P.; Olivier, S.; de Montety, G.; Samuel, C.; Louis-Dreyfus, A.; Tesio, L. Behavioral assessment of unilateral neglect: Study of the psychometric properties of the Catherine Bergego Scale. Arch. Phys. Med. Rehabil. 2003, 84, 51–57. https://doi.org/10.1053/apmr.2003.50062.
18. Jacobson, N.S.; Truax, P. Clinical significance: A statistical approach to defining meaningful change in psychotherapy research. J. Consult. Clin. Psychol. 1991, 59, 12–19. https://doi.org/10.1037/0022-006X.59.1.12.
19. Ottenbacher, K.J.; Hsu, Y.; Granger, C.V.; Fiedler, R.C. The reliability of the Functional Independence Measure: A quantitative review. Arch. Phys. Med. Rehabil. 1996, 77, 1226–1232. https://doi.org/10.1016/S0003-9993(96)90184-7.
20. Bruijnen, C.J.W.H.; Dijkstra, B.A.G.; Walvoort, S.J.W.; Budy, M.J.J.; Beurmanjer, H.; De Jong, C.A.J.; Kessels, R.P.C. Psychometric properties of the Montreal Cognitive Assessment (MoCA) in healthy participants aged 18–70. Int. J. Psychiatry Clin. Pract. 2020, 24, 293–300. https://doi.org/10.1080/13651501.2020.1746348.
21. Fox-Fuller, J.T.; Ngo, J.; Pluim, C.F.; Kaplan, R.I.; Kim, D.H.; Anzai, J.A.U.; Yucebas, D.; Briggs, S.M.; Aduen, P.A.; Cronin-Golomb, A.; et al. Initial investigation of test–retest reliability of home-to-home teleneuropsychological assessment in healthy, English-speaking adults. Clin. Neuropsychol. 2022, 36, 2153–2167. https://doi.org/10.1080/13854046.2021.1954244.
22. Beninato, M.; Gill-Body, K.M.; Salles, S.; Stark, P.C.; Black-Schaffer, R.M.; Stein, J. Determination of the minimal clinically important difference in the FIM instrument in patients with stroke. Arch. Phys. Med. Rehabil. 2006, 87, 32–39. https://doi.org/10.1016/j.apmr.2005.08.130.
23. Hsieh, Y.W.; Wang, C.H.; Wu, S.C.; Chen, P.C.; Sheu, C.F.; Hsieh, C.L. Establishing the minimal clinically important difference of the Barthel Index in stroke patients. Neurorehabil. Neural Repair 2007, 21, 233–238. https://doi.org/10.1177/1545968306294729.
24. Wu, C.Y.; Hung, S.J.; Lin, K.C.; Chen, K.H.; Chen, P.; Tsay, P.K. Responsiveness, minimal clinically important difference, and validity of the MoCA in stroke rehabilitation. Occup. Ther. Int. 2019, 2019, 2517658. https://doi.org/10.1155/2019/2517658.
25. Lindvall, E.; Abzhandadze, T.; Quinn, T.J.; Sunnerhagen, K.S.; Lundström, E. Is the difference real, is the difference relevant: The minimal detectable and clinically important changes in the Montreal Cognitive Assessment. Cereb. Circ. Cogn. Behav. 2024, 6, 100222. https://doi.org/10.1016/j.cccb.2024.100222.
26. Yang, C.M.; Wang, Y.C.; Lee, C.H.; Chen, M.H.; Hsieh, C.L. A comparison of test–retest reliability and random measurement error of the Barthel Index and Modified Barthel Index in patients with chronic stroke. Disabil. Rehabil. 2022, 44, 2099–2103. https://doi.org/10.1080/09638288.2020.1814429.
27. Rorden, C.; Karnath, H.O. A simple measure of neglect severity. Neuropsychologia 2010, 48, 2758–2763. https://doi.org/10.1016/j.neuropsychologia.2010.04.018.
